# POSTN^+^ CAFs facilitate gastric cancer peritoneal metastasis by promoting ICAM-1-dependent tumor cell adhesion and CD8^+^ T-cell exhaustion

**DOI:** 10.3389/fimmu.2026.1796080

**Published:** 2026-06-10

**Authors:** Xin Liu, Qianze Yao, Zhihui Wang, Peiyao Wang, Zichao Zhang, Zexuan Shen, Shilong Li, Yongjia Yan, Weihua Fu

**Affiliations:** 1 Department of General Surgery, Tianjin Medical University General Hospital, Tianjin, China; 2 Department of Gastrointestinal Surgery, The Central Hospital of Wuhan, Tongji Medical College, Huazhong University of Science and Technology, Wuhan, Hubei, China; 3 Department of General Surgery, The First Hospital of Tsinghua University, Beijing, China

**Keywords:** cancer associated fibroblasts, gastric cancer, immunotherapy, T cell, tumor microenvironment

## Abstract

**Background:**

Periostin (POSTN), an extracellular matrix protein secreted by cancer-associated fibroblasts (CAFs), critically shapes the tumor microenvironment. While POSTN contributes to tumor progression and immune regulation by modifying the microenvironment, its direct influence on T-cell activity and antitumor immunity remains unclear.

**Methods:**

In this study, single-cell and bulk RNA sequencing datasets were mined and combined with multiplex fluorescence immunohistochemistry to elucidate the role of POSTN^+^ CAFs. Primary CAFs were isolated and fibroblast lines with elevated POSTN expression were established to assess their direct impact on T-cells through functional assays. Transcriptome sequencing identified downstream regulatory pathways in POSTN^+^ CAFs. A mouse peritoneal metastasis model was used to evaluate the therapeutic potential of targeting POSTN^+^ CAFs in combination with immunotherapy.

**Results:**

Integration of single-cell transcriptomic datasets enabled the construction of a predictive model for gastric cancer peritoneal metastasis (GCPM) based on T-cell exhaustion-associated genes. POSTN was found to be primarily expressed by CAFs in GCPM tissues, with elevated levels correlating with poor clinical outcomes. Functionally, POSTN^+^ CAFs promoted adhesion and spheroid formation by autocrine activation of the AKT-NF-κB-ICAM-1 signaling pathway, facilitating peritoneal dissemination. Co-culture and *in vivo* experiments suggested that POSTN^+^ CAFs may promote CD8^+^ T-cell exhaustion via ICAM-1-ETV3 signaling, resulting in diminished effector function and cytokine production. Inhibiting POSTN downstream signaling with the integrin receptor antagonist Cilengitide, especially together with immune checkpoint blockade (anti-PD-1 and anti-CTLA-4), markedly reduced T-cell exhaustion and suppressed GCPM.

**Conclusion:**

Our results indicate that POSTN^+^ CAFs are pivotal in maintaining an immunosuppressive microenvironment in GCPM. POSTN may facilitates CAFs-tumor and CAFs-T-cell interactions through ICAM-1, thereby promoting metastasis and promoting immune evasion. Targeting the POSTN-ICAM-1 axis may provide a therapeutic approach to enhance immunotherapy efficacy and enhance immunotherapy outcomes in patients with GCPM.

## 1 Introduction

Gastric cancer (GC) is a highly aggressive malignancy, and peritoneal dissemination represents the most frequent pattern of recurrence and metastasis in its advanced stages (1, 2). Peritoneal metastasis (PM) occurs in approximately half of all distant metastasis cases in patients with GC, who consequently face a dismal prognosis with a five-year survival rate below 2% (3–5). Although immunotherapy has substantially improved clinical outcomes for some patients, many remain unresponsive to immune checkpoint inhibitors (ICIs) (6, 7). Therefore, elucidating novel resistance mechanisms and identifying reliable biomarkers or rational combination strategies are essential for extending therapeutic benefit.

PM is primarily driven by endogenous cellular mechanisms, cancer cell adhesion to the peritoneal surface, and the unique tumor microenvironment of the peritoneal cavity (8, 9). Targeting these pathways could yield therapeutic strategies that reduce the incidence and mortality of PM in high-risk primary patients with GC. The tumor immune microenvironment itself is characterized by considerable spatial and functional heterogeneity (10). This immunosuppressive landscape, shaped by diverse inhibitory cells and molecular networks, restricts the universal efficacy of ICIs and complicates patient stratification and outcome prediction (11–13). Modulating these immunosuppressive factors may therefore help overcome current therapeutic limitations, potentially improving patient responses to immunotherapy and extending survival. Recent studies highlight T-cell exhaustion as a central challenge in cancer immunotherapy (14). Through integrating single-cell data and constructing predictive models, we identified Periostin+ (POSTN) CAFs as a potential mediator of T-cell exhaustion, which could underlie the failure of immune therapies. POSTN is a matricellular protein that binds extracellular matrix (ECM) components and cell-surface integrin receptors, thereby mediating cell-cell and cell-matrix interactions and regulating diverse cellular functions such as adhesion, migration, and survival (15). It has been demonstrated that POSTN is predominantly localized within CAFs (16, 17). In cervical squamous cell carcinoma, POSTN+ CAFs compromise lymphatic endothelial barrier function, weakening its restraint on tumor cells and facilitating their entry into the lymphatic system to promote lymph node metastasis (18).

This study provides the first evidence that POSTN+ CAFs contribute critically to T-cell exhaustion, potentially driving the progression of PM in GC. Mechanistically, POSTN+ CAFs secrete POSTN to activate the AKT-NF-κB signaling axis, thereby increasing ICAM-1 expression and enhancing tumor cell adhesion and spheroid formation. Furthermore, ICAM-1 from POSTN+ CAFs triggers ETV3 transcription factor activation in CD8+ T-cells, upregulating genes linked to T-cell exhaustion. Collectively, these findings uncover a previously unknown role for the POSTN+ CAFs-ICAM1-CD8+ T-cell signaling axis in gastric cancer peritoneal metastasis, suggesting POSTN+ CAFs as a promising therapeutic target in this setting.

## 2 Materials and methods

### 2.1 Clinical patient samples

GC tissues and matched adjacent normal tissues were collected from patients undergoing gastrectomy at Tianjin Medical University General Hospital for the isolation of primary CAFs and normal fibroblasts (NFs). Gastric cancer peritoneal metastasis (GCPM) tissues were obtained via laparoscopic biopsy to evaluate POSTN expression. Diagnoses of GC and GCPM were established through preoperative imaging and confirmed by postoperative pathological examination. All tissue specimens were either snap-frozen in liquid nitrogen or formalin-fixed for subsequent analysis. The study received approval from the Ethics Committee of Tianjin Medical University General Hospital (IRB2025-YX-252-01) and adhered to the principles of the Declaration of Helsinki. Written informed consent was provided by all patients before sample collection.

### 2.2 Cell culture

The human GC cell lines AGS, HGC27, MKN45, NCI-N87, SNU216, and KATO III were purchased from ATCC, while the murine NIH 3T3-L1 and YTN16 cell lines were obtained from the Institute at Tianjin Medical University General Hospital. Cells were maintained in either Dulbecco’s modified Eagle’s medium (DMEM, Gibco) or RPMI 1640 medium (Gibco), each supplemented with 10% fetal bovine serum (Gibco) and 1% penicillin/streptomycin (Gibco). All cultures were incubated at 37 °C in a humidified atmosphere of 5% CO_2_.

### 2.3 Bioinformatic analysis

The methodology for identifying T-cell exhaustion-related signature genes and constructing the prognostic model has been detailed previously (19). Briefly, scRNA-seq datasets were acquired from the GEO database (GSE239676, GSE163558). Single-cell RNA sequencing data from primary gastric cancer and peritoneal metastasis samples were obtained from publicly available datasets as described above. The analyzed cohort included primary tumor lesions and peritoneal metastatic samples from gastric cancer patients, and the corresponding clinical information can be accessed from the original published datasets. Quality control was performed using the Seurat package (v4.3). Cells with fewer than 200 detected genes, excessive mitochondrial gene expression (>20%), or potential doublets were excluded from downstream analyses. To minimize batch effects among different samples, data integration and batch correction were conducted using the Harmony algorithm integrated within the Seurat workflow. Samples with incomplete information were excluded, after which expression data were normalized (TPM, log2-transformed) and batch-corrected using the ComBat algorithm. Cell-type annotation was performed based on canonical marker genes combined with previously reported cell lineage signatures. Specifically, epithelial cells were identified by EPCAM and KRT18 expression; fibroblasts by COL1A1, COL1A2, DCN, and FAP; T cells by CD3D and CD3E; macrophages by LYZ and CD68; and endothelial cells by PECAM1 and VWF. CAF subsets were further characterized according to established transcriptional programs, including myCAF-associated markers (ACTA2, TAGLN), inflammatory CAF-related markers (CXCL12, IL6), and antigen-presenting CAF markers (HLA-DRA, CD74). T-cell exhaustion-related genes were collected from GeneCards (https://www.genecards.org/), while immunotherapy response-related genes and differentially expressed genes (fold change >1.2, adjusted p <0.05) were derived from the GSE211645, GSE19826, and GSE239676 datasets. Single-sample GSEA (ssGSEA) was implemented via the GSVA package to quantify activity profiles. Differential pathway enrichment between high- and low-risk groups was evaluated with limma across 50 hallmark pathways, and GO analysis of risk subgroups was performed using clusterProfiler (FDR <0.25, |NES| >1). Weighted gene co-expression network analysis (WGCNA) was employed to construct scale-free networks, from which modules most strongly associated with T-cell exhaustion were selected for further investigation. Cohorts from GSE239676 were stratified by the median T-cell exhaustion-related risk score, and survival differences were evaluated using Kaplan-Meier analysis, log-rank tests, and ROC curves with K-fold cross-validation to ensure robustness. The signature’s prognostic independence was assessed via Cox regression, and a nomogram integrating risk scores and clinical features was developed and validated using ROC curves and C-indexes.

### 2.4 CD8^+^ naïve T-cell sorting

Lymphocytes were isolated from C57BL/6 mouse spleens. CD8^+^ T-cells were subsequently sorted using negative magnetic beads (Selleck, China) according to a previously established protocol (20). Cell purity was assessed by flow cytometry using CD3 and CD8 staining. Only CD3^+^CD8^+^ T-cell preparations with purity exceeding 95% were used for subsequent experiments. Purified T-cells were then activated with anti-CD3/CD28 antibodies (2μg/mL) and cultured in RPMI 1640 medium supplemented with 10% fetal bovine serum and IL-2 (10ng/mL).

### 2.5 Apoptosis experiment and CFSE staining

To evaluate T-cell apoptosis and proliferation after co-culture with fibroblasts, CD8^+^ T-cells were incubated with 3T3-L1^POSTN^ cells at a 1:1 ratio under standard conditions. Following the specified co-culture period, CD8^+^ T-cells were carefully collected and analyzed for apoptosis with an Annexin V-APC/7-AAD Apoptosis Detection Kit (Multi-Science, China) according to the manufacturer’s instructions. Fluorescence was measured on a BD FACSCanto II flow cytometer (BD Biosciences, USA), and the resulting data were processed using FlowJo software (version 10.8). The proportions of early apoptotic (Annexin V^+^/7-AAD^-^) and late apoptotic (Annexin V^+^/7-AAD^+^) cells were determined.

For proliferation assessment, freshly isolated CD8^+^ T-cells were stained with CFSE (Beyotime Biotechnology, China) for 10 minutes at 37 °C in darkness. The labeling reaction was stopped by adding an equal volume of FBS, after which cells were washed twice with RPMI-1640 medium. These CFSE-labeled CD8^+^ T-cells were then co-cultured with either 3T3-L1^POSTN^ or control cells as previously described. After 72 hours, cells were harvested and subjected to flow cytometric analysis. Proliferation was assessed by monitoring sequential reduction in CFSE fluorescence intensity in the FITC channel. Unless otherwise specified, direct contact co-culture systems were used in all *in vitro* experiments. All co-culture experiments were conducted using murine-derived cells to minimize potential confounding effects associated with HLA mismatch or allogeneic immune activation.

### 2.6 Immunohistochemical staining

Human GC tissues, GCPM tissues, and murine peritoneal metastatic tumor samples were fixed in 10% neutral-buffered formalin for 48 hours, dehydrated through a graded ethanol series, cleared in xylene, and embedded in paraffin. Sections of 5 μm thickness were cut, mounted on charged glass slides, deparaffinized in xylene, and rehydrated through descending ethanol concentrations to distilled water. For antigen retrieval, the sections were immersed in EDTA buffer (pH 9.0) and heated in a microwave oven for 15 minutes. Endogenous peroxidase activity was quenched by incubation with 3% hydrogen peroxide for 30 minutes at room temperature, and nonspecific binding sites were blocked with 5% bovine serum albumin (BSA) or normal goat serum for an additional 30 minutes. The slides were then incubated overnight at 4 °C with primary antibodies diluted in antibody diluent. Following three washes with PBS, the sections were incubated with a biotinylated secondary antibody for 1 hour at 37 °C and subsequently with a streptavidin-HRP complex. Immunoreactive signals were developed using a 3,3′-diaminobenzidine (DAB) substrate kit (ZSGB-BIO, China) and counterstained with hematoxylin. Finally, the slides were dehydrated, cleared, and mounted with neutral resin. Images were acquired using a Leica DM2000 microscope (Leica Microsystems, Germany). All antibodies used are listed in Supplemental **Supplementary Table 2**.

### 2.7 Immunofluorescence staining

IF on tissue sections followed a protocol similar to that used for immunohistochemical staining. Following incubation with the primary antibody, the corresponding fluorescent secondary antibody was applied at 37 °C for one hour. Nuclei were then counterstained with DAPI (Beyotime, China). For cell samples, IF staining required fixation with 4% paraformaldehyde and permeabilization with 1% Triton X-100. After blocking with 5% BSA, the samples were incubated with the appropriate primary and fluorescent secondary antibodies. All subsequent steps matched the procedure used for tissue sections.

### 2.8 Primary normal fibroblasts and CAFs isolation

Primary NFs and CAFs were isolated from GC tumor tissues and their matched normal adjacent tissues. Freshly harvested tissues were rinsed in ice-cold PBS containing 5% penicillin/streptomycin for 30 minutes. Following two washes, the tissues were minced into 1–2 mm fragments and digested in a solution of 1 mg/mL collagenase I and 1 mg/mL collagenase IV at 37 °C for 1.5 hours. The digestion was halted by adding an equal volume of complete culture medium, and the resulting cell suspension was filtered through a 70 μm mesh. After centrifugation, the isolated cells were resuspended in DMEM supplemented with 20% FBS and left undisturbed for 96 hours. Non-adherent cells were subsequently removed, and the remaining adherent fibroblasts were maintained in culture. Following 3–4 passages, fibroblast purity was evaluated by immunofluorescence staining using fibroblast markers (α-SMA, FAP). Only cultures with a fibroblast purity >90% were used for subsequent experiments.

### 2.9 qRT-PCR

Total RNA was extracted from cells with TRIzol reagent (Solarbio, China). RNA concentration was quantified using a Nanodrop, and reverse transcription was carried out with the Quantitect Reverse Transcription Kit (ACCURATE BIOTECHNOLOGY(HUNAN) CO., LTD, ChangSha, China). Quantitative RT-PCR was subsequently performed using the SYBR Green Premix Pro Taq HS qPCR Kit (Vazyme, China) in accordance with the manufacturer’s instructions. GAPDH served as the internal reference gene for semi-quantitative analysis. The sequences for all primers used in this study are provided in Supplemental **Supplementary Table 1**.

### 2.10 Western blotting

The cells were washed with ice PBS and lysed using RIPA buffer (Beyotime, China) supplemented with protease inhibitors (Beyotime, China) and phosphatase inhibitors (Beyotime, China). Protein concentration was quantified using a BCA assay (Solarbio, China). Western blotting was conducted as previously described (21). The antibodies used are listed in Supplemental **Supplementary Table 2**.

### 2.11 Cell adhesion experiments

Following the designated pretreatments, 3T3-L1 cells were plated in 12-well plates to allow adhesion. After a 2 h incubation at 37 °C or 1 h of shaking at 70 rpm, the cells were washed with PBS, fixed with paraformaldehyde for 15 min, and stained with 0.1% crystal violet for 20 min at room temperature. Excess dye was then removed by PBS washing before the cells were imaged under a microscope. Cell numbers were counted to evaluate adhesion.

For the fibroblast-tumor cell adhesion assay, tumor cells were fluorescently labeled with FITC, while fibroblasts were counterstained with PKH26 according to the manufacturer’s instructions. After labeling, tumor cells were seeded onto 3T3-L1 cells monolayers and incubated under standard culture conditions to permit adhesion. Non-adherent cells were subsequently removed by gentle PBS washing, and adherent cells were visualized using fluorescence microscopy. In parallel, fibroblast-tumor cell spheroid formation assays were performed using ultra-low attachment plates. Following fluorescent labeling, tumor cells and fibroblasts were combined at a 2:1 ratio (2 × 10^5^ tumor cells and 1 × 10^5^ fibroblasts per well) and seeded into 12-well non-tissue plates. These mixed cultures were maintained for 24 h before spheroid formation was assessed microscopically. The average spheroid diameter was maintained approximately 100–150 μm to minimize potential hypoxia-related effects on ICAM-1 expression. Images were acquired, and adhesion efficiency was quantified based on the number of tumor cells incorporated into fibroblast-containing spheroids.

### 2.12 Transcriptome sequencing (RNA-seq)

Total RNA was extracted from co-cultured CD8^+^ T-cells with TRIzol reagent (Invitrogen, USA) according to the manufacturer’s protocol. RNA concentration and integrity were assessed using a TBS380 fluorometer. Majorbio Bio-Pharm Technology Co., Ltd. (Shanghai, China) prepared the libraries and performed paired-end sequencing on the Illumina HiSeq platform using these high-quality RNA samples. Transcript abundance was quantified as transcripts per million reads (TPM). We identified differentially expressed genes (DEGs) between groups with the DESeq2 package, applying thresholds of |log_2_FC| ≥ 1 and a false discovery rate (FDR) ≤ 0.05. Visualization and downstream analyses were carried out using the integrated tools on Majorbio’s online platform.

### 2.13 Chromatin immunoprecipitation assay

ChIP assays were performed with the SimpleChIP Enzymatic Chromatin IP Kit (CST, USA) following the manufacturer’s protocol (21). Following ultrasonication, chromatin samples underwent immunoprecipitation overnight at 4 °C using anti-ETV3 or control IgG. The purified DNA was then analyzed via RT-qPCR, with enrichment calculated relative to a 2% input control. The corresponding primer sequences are listed in Supplemental **Supplementary Table 3**.

### 2.14 *In vivo* experiments

Female C57BL/6 mice (4 weeks old) were obtained from GemPharmatech (Nanjing, China) and maintained under specific pathogen-free conditions at the Laboratory Animal Center of Tianjin General Surgery Institute. The mice were housed under controlled temperature and a 12-hour light/dark cycle with ad libitum access to food and water. All animal procedures followed institutional guidelines and were approved by the Ethics Committee of Tianjin Medical University General Hospital (IRB2025-DWFL-631). To establish the GCPM model, each mouse received an intraperitoneal injection of 2 × 10^6^ stably transfected 3T3-L1 cells and 5 × 10^6^ YTN16 cells suspended in 1 mL PBS (n = 5 per group). Mice were randomly assigned to experimental groups. Body weight and general condition were monitored every other day for 21 days to assess tumor burden and systemic effects. Peritoneal metastatic burden was evaluated at the experimental endpoint based on the number and size of peritoneal tumor implants. For evaluating the immunotherapy-sensitizing effect of POSTN/integrin pathway inhibition, mice were randomly assigned to groups receiving: Control (vehicle); Anti-PD-1 antibody (10 mg/kg) + Anti-CTLA-4 antibody (10 mg/kg); or Anti-PD-1 (10 mg/kg) + Anti-CTLA-4 (10 mg/kg) + Cilengitide (5 mg/kg). All treatments were administered intraperitoneally every other day throughout the study. At the endpoint, peritoneal lavage fluid and tumor implants were collected for subsequent analyses, including flow cytometry and immunohistochemistry, to assess immune cell infiltration and therapeutic efficacy.

### 2.15 Flow cytometry analysis

In this study, the specific steps for flow cytometry were performed according to previous studies (21). After treatment, cells were harvested, washed with PBS, and blocked with an antibody. Surface staining was conducted prior to membrane permeabilization and intracellular antibody staining, followed by fixation. Data acquisition was performed on a BD FACSCanto II flow cytometer and analyzed with FlowJo 10.8.1. All antibodies are listed in Supplemental **Supplementary Table 2**.

### 2.16 Ethics approval and consent to participate

This study was approved by the Ethics Committee of Tianjin Medical University General Hospital (IRB2025-YX-252-01) and conducted in accordance with the principles of the Declaration of Helsinki. Written informed consent was obtained from all patients prior to sample collection. All animal experiments were approved by the Ethics Committee of Tianjin Medical University General Hospital (IRB2025-DWFL-631).

### 2.17 Statistical analysis

All statistical analyses were performed with GraphPad Prism 9 and R software (version 4.2.2). Normality of data distribution was assessed using the Shapiro–Wilk test. Differences between two groups were evaluated using two-tailed unpaired Student’s t-tests, while comparisons among multiple groups were performed using one-way or two-way analysis of variance (ANOVA) as appropriate. Tukey’s multiple comparisons test was applied as the *post-hoc* analysis following one-way ANOVA, while Bonferroni’s *post-hoc* test was used following two-way ANOVA to determine specific group differences. Data are expressed as mean ± standard deviation (S.D.). All experiments were independently repeated at least three times, and technical replicates were included where applicable. Survival analysis utilized the Kaplan-Meier method, with differences between survival curves assessed by the log-rank test. A P-value below 0.05 was considered statistically significant. All details regarding replicates, statistical tests, and *post-hoc* analyses are also indicated in the figure legends where applicable.

## 3 Results

### 3.1 Identification of POSTN as central players in the immunoregulatory network of gastric cancer peritoneal metastasis

**Figure 1:**
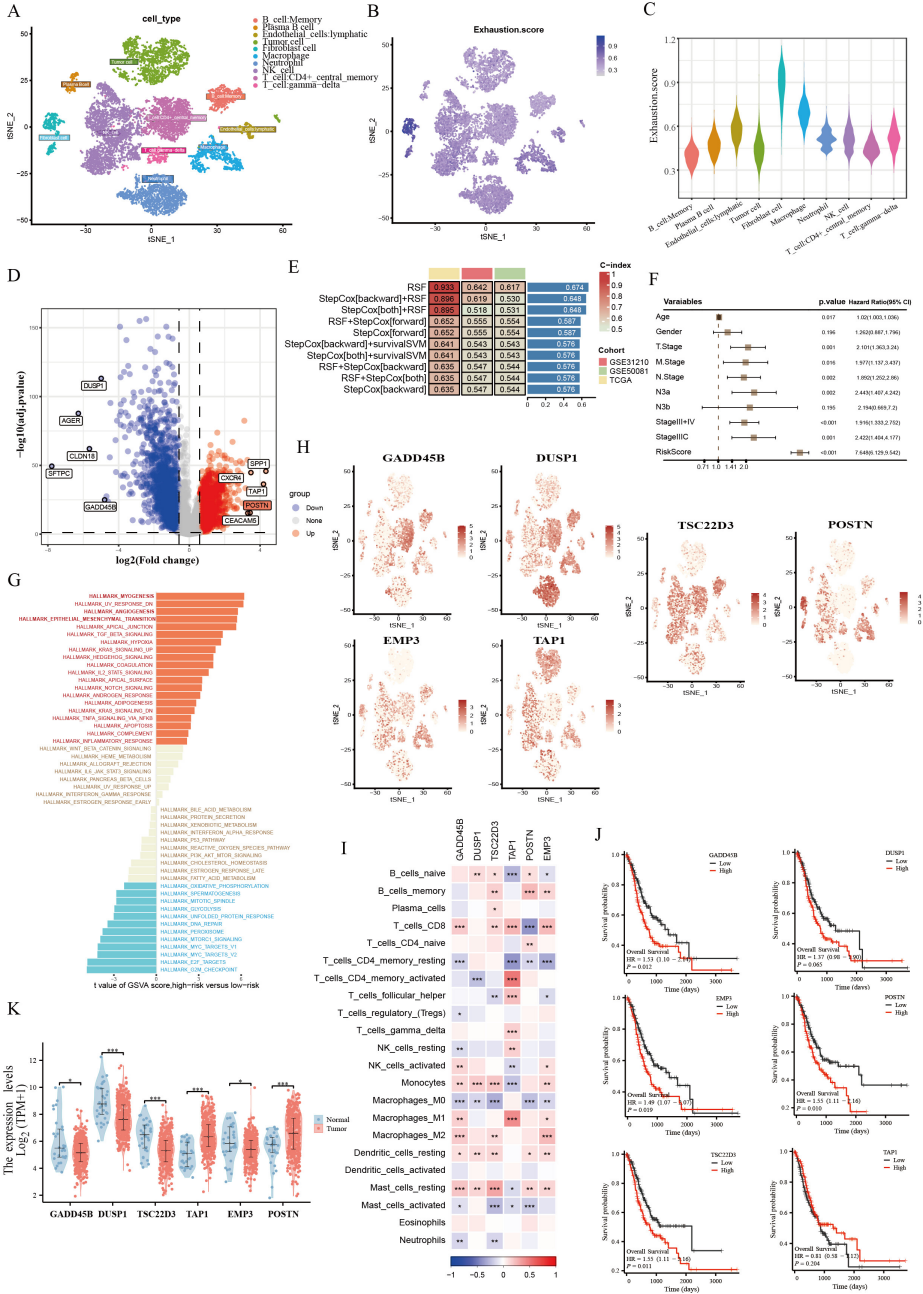
Integrative machine learning analysis uncovers exhaustion-associated mechanisms and develops a prognostic model for peritoneal metastasis in GC. **(A)** Cell type classification of single-cell RNA-seq data was conducted according to the expression patterns of canonical marker genes. **(B)** The activity scores of exhaustion-associated genes were calculated and visualized for each single cell. **(C)** Violin plots illustrating the expression patterns of exhaustion-associated genes among different cell types. **(D)** Volcano plot illustrating differential gene expression between peritoneal metastatic and primary GC samples, with the top five genes labeled. **(E)** A total of 101 predictive models were developed using a rigorous 10-fold cross-validation framework, with the top ten predictive models shown. **(F)** Univariate analysis of clinical characteristics along with predictive model regarding OS. **(G)** Assessment of the correlation between risk scores derived from the GSVA-based exhaustion model and the activity of representative pathways. **(H)** GADD45B, DUSP1, TSC22D3, POSTN, EMP3 and TAP1 were analyzed by scRNA-seq. **(I)** Correlation analysis was performed to assess the relationship between the six model genes and the abundance of immune cells in the tumor microenvironment. **(J)** Prognostic analysis of the six genes used in the predictive model in GC patients. **(K)** Comparison of the expression levels of the six model genes between GC tissues and paired adjacent normal tissues in the TCGA cohort. Statistical significance was determined using a two-tailed unpaired Student’s t-test. Data are presented as mean ± standard deviation (S.D.). Statistical methods for each analysis are indicated where applicable. *P < 0.05; **P < 0.01; ***P < 0.001. GC, gastric cancer; OS, overall survival; GSVA, Gene Set Variation Analysis; scRNA-seq, single-cell RNA sequencing.

By integrating T-cell exhaustion-related genes, immunotherapy response signatures from GSE211645, and differentially expressed genes from two independent gastric cancer datasets (GSE19826, GSE239676), we identified 33 overlapping genes potentially involved in immune regulation and metastatic progression. To determine their cellular specificity, we analyzed the expression patterns of these 33 intersecting genes using single-cell RNA sequencing data from GCPM (GSE239676 and GSE163558), which revealed their distribution across distinct cellular subsets within the tumor microenvironment (**Figures 1A–C**). These genes showed markedly elevated expression in CAFs) and a subset of macrophages, suggesting these populations may mediate stromal remodeling and immune suppression in GCPM (**Figures 1B, C**). Furthermore, these genes were significantly dysregulated in GCPM compared with primary gastric cancer lesions (**Figure 1D**). We implemented a comprehensive machine learning framework incorporating 101 algorithms to develop prognostic models, among which the random survival forest (RSF) model demonstrated optimal predictive performance (**Figure 1E**). Multivariate survival analysis confirmed that the RSF-derived risk score served as an independent prognostic indicator for GCPM patients (**Figure 1F**; **Supplementary Figures 1A, B**). Functional enrichment analysis using GSVA indicated that the high-risk subgroup exhibited enhanced activity in epithelial-mesenchymal transition (EMT), angiogenesis, and myogenesis pathways, all processes linked to tumor invasion and metastasis (**Figure 1G**). Additionally, high-risk gastric cancer patients showed significantly elevated immune and stromal scores along with increased expression of multiple immune checkpoint molecules, suggesting an immune-exhausted microenvironment (**Supplementary Figures 1C, D**). We further examined the expression and cellular distribution of the six model-constructing genes in the single-cell tumor microenvironment (**Figure 1H**). All six genes correlated to varying degrees with immune cell populations (**Figure 1I**). Integrated analysis of gene expression and survival data indicated that only POSTN was markedly upregulated in gastric cancer and significantly associated with poor prognosis (**Figures 1J, K**; **Supplementary Figure 1E**). Single-cell transcriptomic analysis further revealed that POSTN expression was predominantly restricted to CAF populations, implying that its pro-metastatic and immunomodulatory roles in GCPM are largely CAF-mediated (**Supplementary Figure 1F**). These findings establish POSTN as a key CAF-derived gene linked to immune regulation, metastatic potential, and unfavorable prognosis in GCPM.

### 3.2 Spatial localization and clinical relevance of POSTN^+^ CAFs reveal their immunoregulatory role in gastric cancer peritoneal metastasis

**Figure 2:**
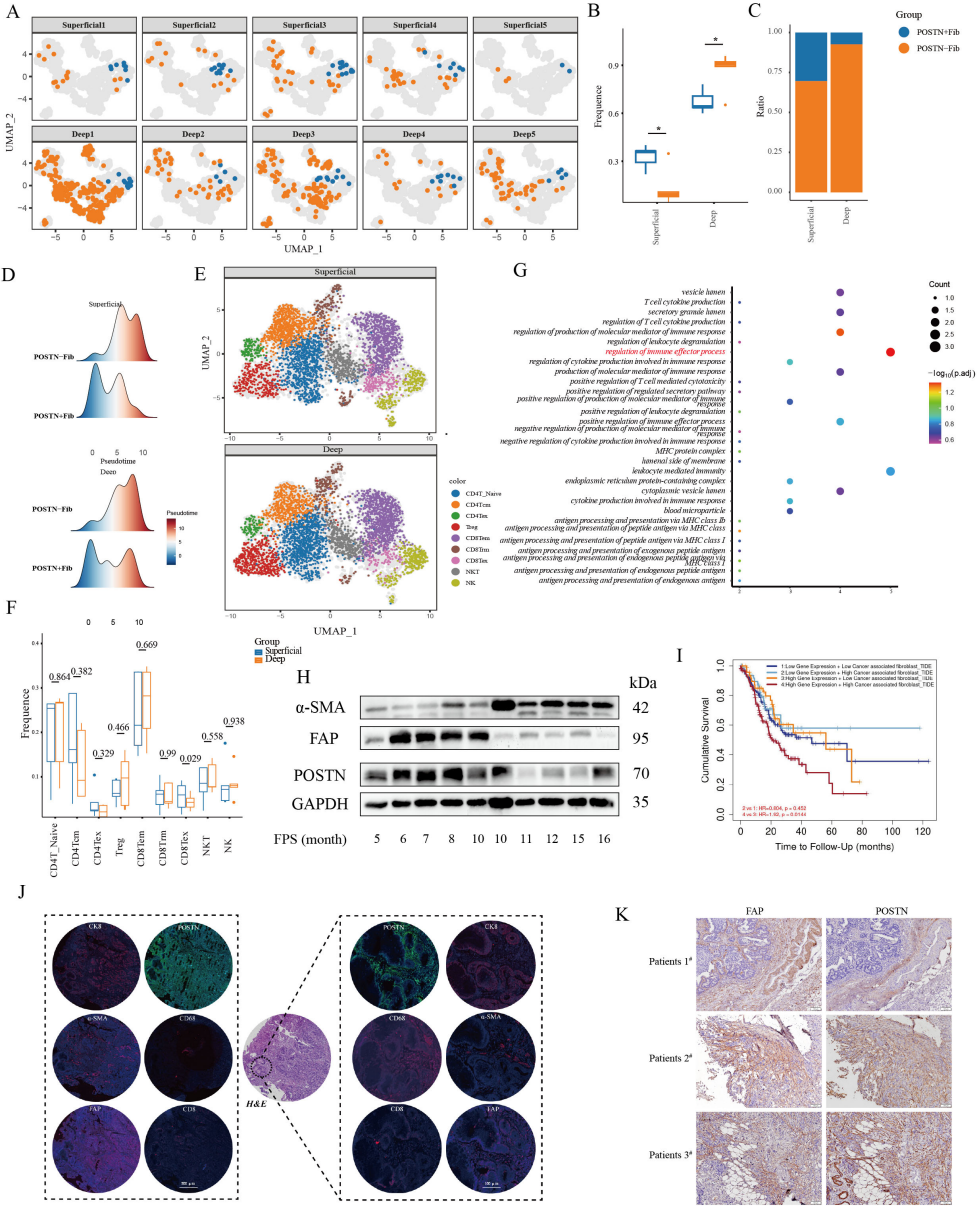
POSTN^+^ CAFs are significantly associated with peritoneal metastasis and poor prognosis in patients with GC. **(A)** Representative distribution of POSTN^+^ CAFs across different invasion depths of primary lesions in GCPM (GSE167297, *n* = 5 per group). **(B, C)** Frequency **(B)** and ratio **(C)** of POSTN^+^ CAFs and POSTN^-^ CAFs in GCPM. **(D)** Pseudotime trajectory analysis illustrating the distinct developmental dynamics and transcriptional states of POSTN^+^ versus POSTN^-^ CAFs in superficial and deep tissues. **(E, F)** Analysis of the distribution and relative proportions of immune cell populations across different invasion depths of primary lesions in GC. **(G)** Functional enrichment analysis illustrating the biological processes and signaling pathways associated with POSTN^+^ CAFs. **(H)** Western blot analysis of FAP, αSMA, and POSTN expression in primary CAFs isolated from GC patients with postoperative PM. Representative results from three independent experiments are shown. n = 3 per group. **(I)** Prognostic impact of POSTN expression and CAFs infiltration in GC patients analyzed using the TIMER 2.0 database. **(J)** Multi-immunostaining images of CK8 (red), POSTN (green), αSMA (red), FAP (red), CD68 (red) and CD8 (red) in tumor tissues of patients with GC from TJMUGH (scale bars: 100 µm). **(K)** Representative IHC staining images for POSTN and FAP expression level in GCPM samples (*n* = 3, scale bars: 100 µm). Data are presented as mean ± standard deviation. *P < 0.05. POSTN, periostin; GC, gastric cancer; GCPM, gastric cancer peritoneal metastasis; IHC, immunohistochemistry; TJMUGH, Tianjin Medical University General Hospital; TIMER, Tumor Immune Estimation Resource; OS, overall survival; CAFs, cancer associated fibroblasts; FPS, Progression-free survival.

To elucidate the functional localization of POSTN in gastric cancer peritoneal metastasis (GCPM), we analyzed its expression across various metastatic sites. POSTN expression was significantly higher in peritoneal metastases than in primary tumors (**Supplementary Figure 2A**). Consistent with this, analysis of our previously established orthotopic mouse model of gastric cancer with peritoneal dissemination confirmed elevated POSTN expression in metastatic nodules (**Supplementary Figure 2B**) (21). A strong positive correlation emerged between POSTN expression and fibroblast infiltration in GC (r = 0.816) (**Supplementary Figure 2C**). Single-cell transcriptomic profiling of superficial and deep GC tissues indicated a greater proportion of POSTN^+^ CAFs in the superficial compartments (**Figures 2A–C**). Pseudotime analysis further revealed transcriptional heterogeneity and dynamic cellular states within POSTN-enriched CAF populations, indicating functional heterogeneity within this population (**Figure 2D**). When comparing immune infiltration between superficial and deep tissues, we observed more pronounced T-cell exhaustion in the superficial compartments (**Figures 2E, F**). POSTN^+^ CAFs in these regions also exhibited marked immunoregulatory functions, implying a role in local immune suppression (**Figure 2G**). To assess the prognostic relevance of POSTN in GCPM, we analyzed its expression in tumor samples from patients stratified by time to PM recurrence. Higher POSTN expression correlated with shorter recurrence intervals, suggesting its potential as a predictive biomarker for aggressive peritoneal dissemination (**Figure 2H**). In agreement with our experimental data, analysis of public datasets showed that increased POSTN^+^ CAF abundance was associated with poorer prognosis in GC patients (**Figure 2I**), while infiltration by other immune cell types showed no significant association with outcomes (**Supplementary Figure 2D**). Finally, IF and IFC analyses validated POSTN localization within GC tissues and confirmed its upregulation in peritoneal metastatic lesions (**Figures 2J, K**). These findings collectively underscore a critical role for POSTN^+^ CAFs in driving PM in GC.

### 3.3 POSTN^+^ CAFs induce CD8^+^ T-cell exhaustion and suppress effector function through direct cell-cell interactions

**Figure 3:**
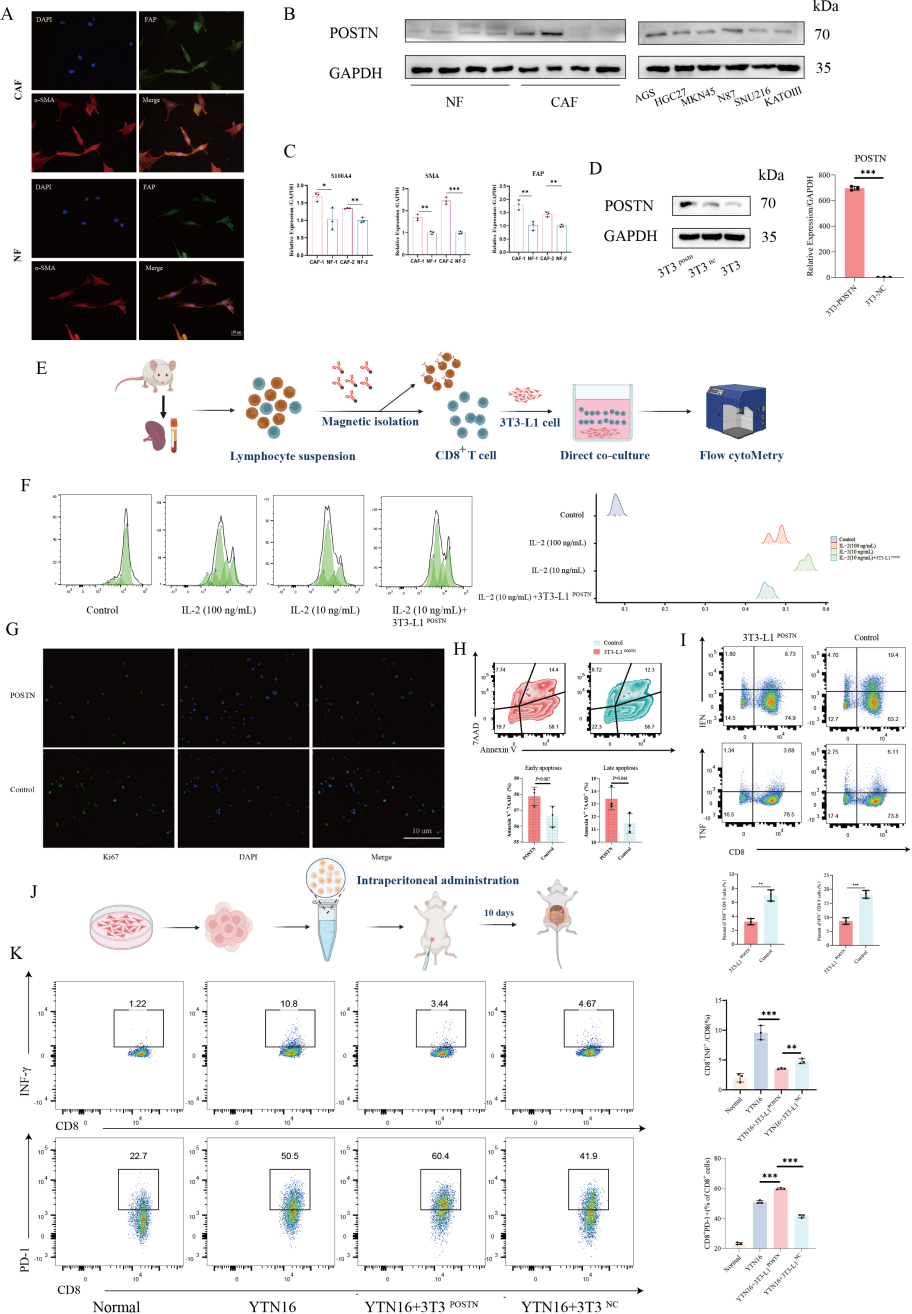
POSTN^+^ CAFs induced CD8^+^ T-cell exhaustion *in vitro* and *in vivo*. **(A)** Multi-immunostaining of FAP, αSMA, and POSTN in primary CAFs and NFs (scale bars: 100 µm). **(B)** Western blot analysis of POSTN in CAFs, NFs and GC cell lines. Representative results from three independent experiments are shown. **(C)** qRT-PCR analysis of S100A4, SMA and FAP expression level in CAFs and NFs. **(D)** Construction of stable POSTN up-regulated 3T3-L1 cell lines. *n* = 3 per group. **(E)** Schematic illustration of CD8^+^ T-cell isolation from spleens of C57BL/6 mice and the direct co-culture system with 3T3-L1 cells. CD8^+^ T cells were activated with anti-CD3/CD28 antibodies and directly co-cultured with 3T3-L1 cells at a 1:1 ratio (1 × 10^5^ cells each) for 72 h prior to subsequent analyses. **(F)** Proliferation of CD8^+^ T-cells assessed by CFSE dilution after co-culture with POSTN-overexpressing 3T3-L1^POSTN^ cells. *n* = 3 per group. **(G)** Immunofluorescence staining of Ki67 expression in CD8^+^ T-cells following co-culture with POSTN-overexpressing 3T3-L1 cells (scale bars: 10 µm). *n* = 3 per group. **(H)** Apoptosis analysis of CD8^+^ T-cells after co-culture with POSTN-overexpressing 3T3-L1 cells. *n* = 3 per group. **(I)** Representative flow cytometry analysis of cytokines expression of TNF-α and IFN-γ in CD8 ^+^ T-cells. *n* = 3 per group. **(J)** Flowchart illustrating the experimental procedure of intraperitoneal implantation of tumor cells and 3T3-L1 cells. 3T3-L1^POSTN^, 3T3-L1^NC^, and YTN16 cells were expanded in culture, mixed, and intraperitoneally injected into C57BL/6 mice. Ten days after injection, the mice were euthanized, and peritoneal lavage fluid was collected for flow cytometric analysis. **(K)** Representative flow cytometry plots showing PD-1 and IFN-γ expression in CD8^+^ T cells isolated from the peritoneal lavage fluid of mice. *n* = 3 per group. Data are presented as mean ± standard deviation. *P < 0.05; **P < 0.01; ***P < 0.001. POSTN, periostin; GC, gastric cancer; CAFs, cancer associated fibroblasts; NFs, normal fibroblasts; CFSE, carboxyfluorescein succinimidyl ester.

To investigate the expression profile of POSTN within CAFs, primary CAFs and corresponding normal fibroblasts (NFs) were extracted from human GC specimens, enabling a direct comparison of POSTN levels between tumor-associated and normal stromal fibroblasts (**Figures 3A, C**; **Supplementary Figure 3A**). Our results demonstrated that POSTN was markedly upregulated in a subset of CAFs relative to NFs (**Figure 3B**). In contrast, POSTN expression levels were comparatively low in various GC cell lines (**Figure 3B**; **Supplementary Figure 3B**). For subsequent mechanistic studies, we established a sTable 3T3-L1 fibroblast line overexpressing POSTN (3T3-L1^POSTN^), enabling functional evaluation of POSTN^+^ CAF *in vitro* and *in vivo* (**Figure 3D**). Since POSTN by itself did not significantly impact T-cell function or phenotype (**Supplementary Figures 3C, D**), we speculated that POSTN^+^ CAFs might induce T-cell exhaustion by direct interactions or indirect regulatory pathways. CD8^+^ T-cells were purified from mouse spleens via magnetic bead separation, and a contact-dependent co-culture model with 3T3-L1^POSTN^ cells was established to evaluate their impact on T-cell functionality (**Figure 3E**) (22). Co-culture experiments revealed that 3T3-L1^POSTN^ cells markedly inhibited T-cell proliferation, downregulated Ki67 expression, and promoted partial apoptosis (**Figures 3F–H**). Moreover, following co-culture with 3T3-L1^POSTN^ cells, CD8^+^ T-cells exhibited increased expression of immune inhibitory receptors and exhaustion-related transcription factors, accompanied by a reduction in effector cytokine secretion, suggesting a direct role of POSTN^+^ CAF in dampening T-cell effector functions (**Figure 3I**; **Supplementary Figure 3H**). To further confirm the modulator effects of 3T3-L1^POSTN^ cells on T-cell function and phenotype *in vivo*, we constructed a PM model by co-implanting 3T3-L1^POSTN^ cells with the murine GC cell line YTN16 (**Figure 3J**). Our results demonstrated that 3T3-L1^POSTN^ cells inhibited IFN-γ secretion and increased PD-1 expression in CD8^+^ T-cells within the immune landscape of the peritoneal tumor microenvironment, indicating induction of a functionally exhausted phenotype (**Figure 3K**). Furthermore, POSTN^+^ CAFs decreased the frequencies of central memory (Tcm) and effector (Teff) subsets among both CD8^+^ and CD4^+^ T-cells, underscoring their capacity to reshape the tumor immune microenvironment and induce T-cell dysfunction in peritoneal metastases. (**Supplementary Figures 3F, G**). Together, these findings demonstrate that POSTN^+^ CAFs suppress CD8^+^ T-cell proliferation and effector function while promoting T-cell exhaustion, highlighting their critical role in remodeling the immune microenvironment in GCPM.

### 3.4 Autocrine POSTN upregulates ICAM-1 expression and strengthens CAFs adhesive capacity

**Figure 4:**
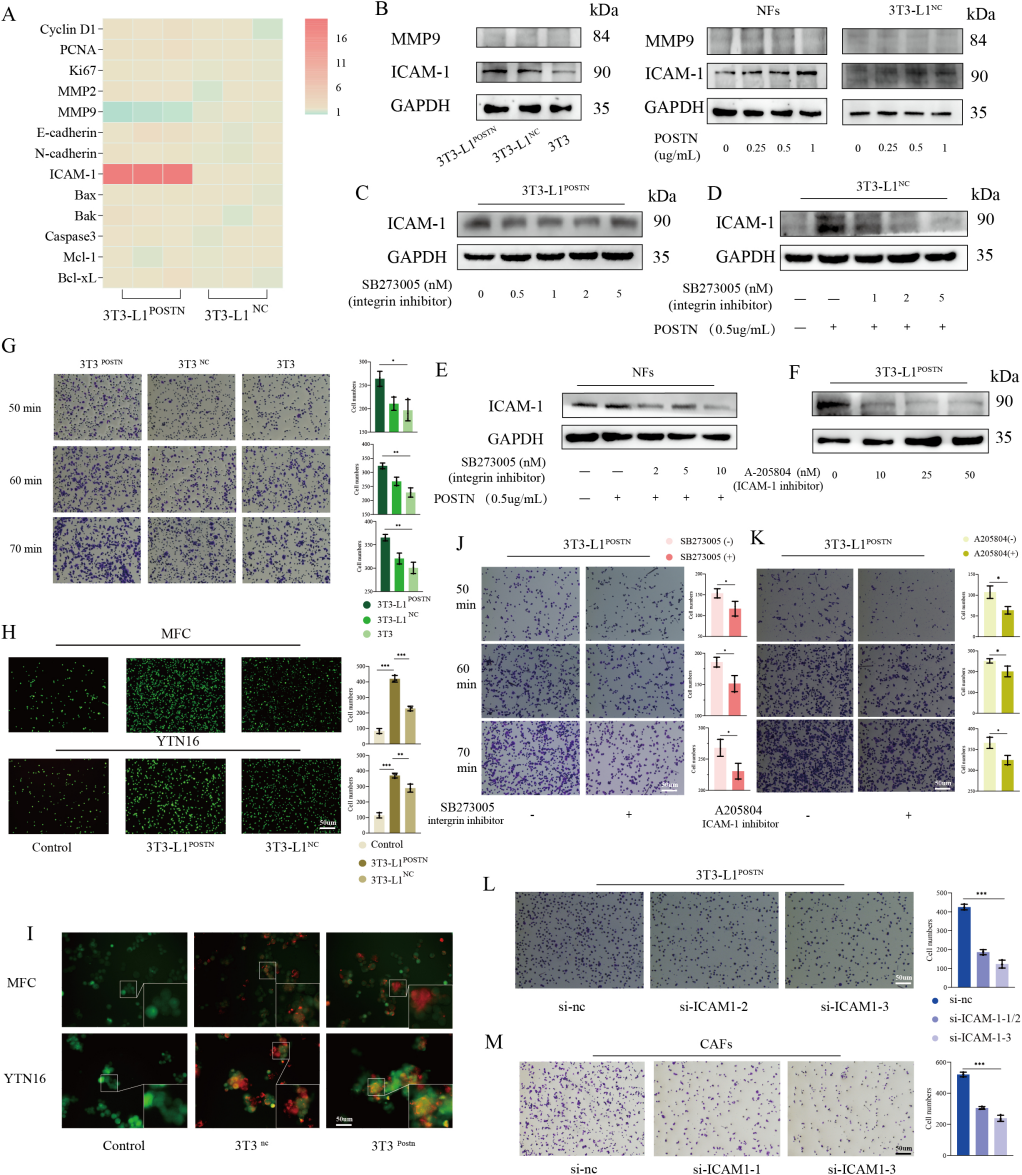
POSTN^+^ CAFs facilitate GC tumor spheroid formation through ICAM-1 dependent regulation. **(A)** qRT-PCR analysis of the mRNA expression levels of proliferation and adhesion markers in 3T3-L1^POSTN^ and 3T3-L1^NC^ cells. **(B)** Western blot analysis showing the protein expression levels of MMP9 and ICAM-1 in 3T3-L1 cells and NFs following treatment with increasing concentrations of POSTN protein. *n* = 3 per group. **(C)** Western blot analysis of ICAM-1 in 3T3-L1^POSTN^ cells after treatment with SB273005. *n* = 3 per group. **(D, E)** Western blot analysis of ICAM-1 in 3T3-L1^NC^ **(D)** and NFs **(E)** cells after treatment with SB273005 and POSTN protein. *n* = 3 per group. **(F)** Western blot analysis of ICAM-1 in 3T3-L1^POSTN^ cells after treatment with A-205804. Representative results from three independent experiments are shown. **(G)** Cell adhesion assay assessing 3T3-L1 cell attachment at 50, 60, and 70 minutes after plating. A total of 1 × 10^5^ cells were seeded into 12-well plates for each assay. *n* = 3 per group. **(H)** Adhesion assay evaluating the attachment of FITC-labeled MFC and YTN16 tumor cells to adherent 3T3-L1^POSTN^ cells. Tumor cells and fibroblasts were co-cultured at a 2:1 ratio (2 × 10^5^ tumor cells and 1 × 10^5^ fibroblasts per well) in 12-well plates for 2 h, followed by PBS washing, fixation, and fluorescence imaging. n = 3 per group. **(I)** Fibroblast–tumor cell adhesion assay assessing the effects of POSTN on cell adhesion. MFC and YTN16 cells were labeled with FITC. 3T3-L1^POSTN^ and 3T3-L1^NC^ cells were labeled with PKH26. **(J)** Cell adhesion assay assessing 3T3-L1 cell attachment after treatment with SB273005. *n* = 3 per group. **(K)** Cell adhesion assay assessing 3T3-L1 cell attachment after treatment with A-205804. *n* = 3 per group. **(L, M)** Cell adhesion assay assessing 3T3-L1^POSTN^ and CAFs cells attachment after knockdown ICAM-1. *n* = 3 per group. Data are presented as mean ± standard deviation. *P < 0.05; **P < 0.01; ***P < 0.001. GC, gastric cancer; POSTN, periostin; GC, gastric cancer; CAFs, cancer associated fibroblasts; NFs, normal fibroblasts; NC, negative control; qRT-PCR, quantitative Real-time polymerase chain reaction; MFC, Mouse forestomach carcinoma.

To investigate whether POSTN affects CAFs in an autocrine manner, we analyzed the phenotype of 3T3-L1^POSTN^ cells. The results showed no significant changes in CAF-associated markers compared with controls (**Supplementary Figure 4A**). By assessing changes in proliferation and adhesion-related markers, we observed that ICAM-1 and MMP9 exhibited significant differential expression (**Figure 4A**). Western blot results demonstrated that ICAM-1 expression was markedly increased in 3T3-L1^POSTN^ cells compared with controls, while MMP9 levels showed no significant difference (**Figure 4B**). Treatment with recombinant POSTN led to a dose- and time-dependent increase in ICAM-1 expression in NFs and 3T3-L1 cells (**Figure 4B**; **Supplementary Figure 4B**). Treatment with the αvβ3/αvβ5 integrin inhibitor SB273005 reduced ICAM-1 expression in 3T3-L1^POSTN^ cells and blocked the upregulation induced by exogenous recombinant POSTN (**Figures 4C–E**; **Supplementary Figure 4C**). Considering that ICAM-1 is a classical adhesion molecule (23), we investigated whether 3T3-L1^POSTN^ cells exhibit enhanced cell-cell adhesive capacity, potentially contributing to tumor cell interaction and metastasis. The results demonstrated that 3T3-L1^POSTN^ cells displayed significantly enhanced substrate adherence and tumor cell-binding ability relative to controls (**Figures 4G–I**; **Supplementary Figure 4D**). SB273005 treatment markedly attenuated the adhesive capacity of 3T3-L1^POSTN^ cells (**Figure 4J**). To clarify whether the enhanced adhesion of 3T3-L1^POSTN^ cells is dependent on ICAM-1, we treated the cells with the ICAM-1 inhibitor A-205804 and employed siRNA-mediated knockdown of ICAM-1 (**Figure 4F**; **Supplementary Figures 4C, E**). The result showed that blockade of ICAM-1, either pharmacologically or through siRNA knockdown, markedly diminished the adhesive capacity of 3T3-L1^POSTN^ cells and CAFs (**Figures 4K–M**; **Supplementary Figure 4F**). Moreover, supplementation with recombinant ICAM-1 protein rescued the impaired adhesive capacity of 3T3-L1^POSTN^ cells caused by SB273005 treatment (**Supplementary Figure 4G**). These results indicate that POSTN enhances the adhesive capacity of CAFs primarily through upregulation of ICAM-1.

### 3.5 POSTN activates the AKT-NF-κB pathway to drive ICAM-1-dependent fibroblast adhesion

**Figure 5:**
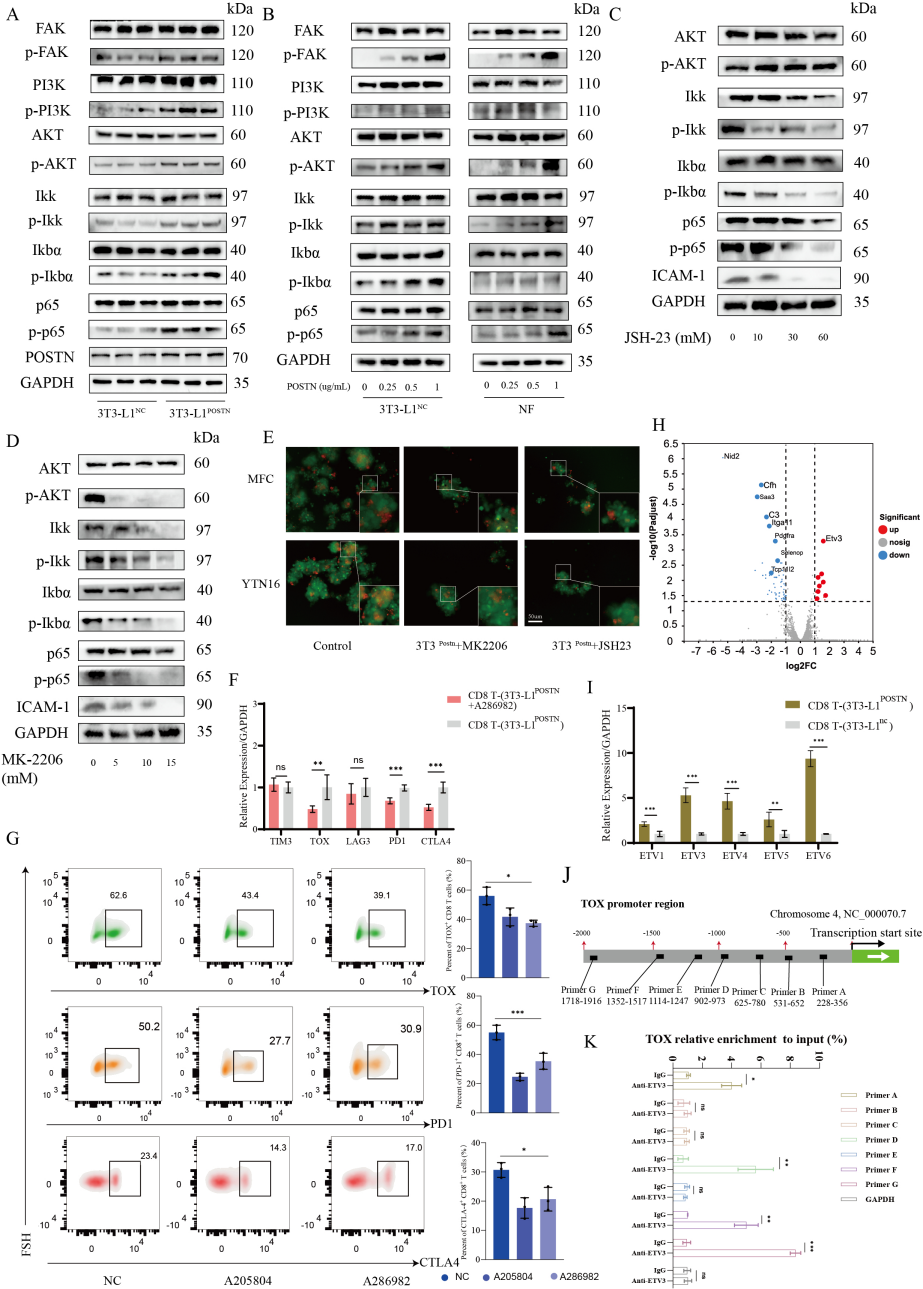
POSTN induces CD8^+^ T-cell exhaustion through regulation of the ICAM-1–ETV3 signaling pathway. **(A)** Western blot analysis showing the AKT-NF-kB pathway in 3T3-L1^POSTN^ and 3T3-L1^NC^ cells. *n* = 3 per group. **(B)** Western blot analysis showing the expression levels of AKT-NF-κB pathway in 3T3-L1 cells and NFs following treatment with increasing concentrations of POSTN protein. *n* = 3 per group. **(C, D)** Western blot analysis showing the expression of ICAM-1 in 3T3-L1^POSTN^ cells following treatment with AKT (MK-2206) and NF-κB (JSH-23) inhibitors. Representative results from three independent experiments are shown. **(E)** Fibroblast–tumor cell adhesion assay assessing the effects of AKT (MK-2206) and NF-κB (JSH-23) inhibitors on cell adhesion. FITC-labeled MFC and YTN16 tumor cells were co-cultured with PKH26-labeled 3T3-L1^POSTN^ cells at a 2:1 ratio (2 × 10^5^ tumor cells and 1 × 10^5^ fibroblasts per well) in 12-well plates for 2 h (scale bars: 100 µm). **(F)** qRT-PCR analysis showing the mRNA expression levels of exhaustion-associated genes in CD8^+^ T cells following co-culture with 3T3-L1^POSTN^ cells treated with A286982 (LFA-1/ICAM-1 inhibitor). *n* = 3 per group. **(G)** Representative flow cytometry analysis of cytokines expression of TOX, PD-1, and CTLA-4 in CD8^+^ T-cells following co-culture with 3T3-L1^POSTN^ cells treated with A205804 and A286982. *n* = 3 per group. **(H)** Transcriptomic analysis identifying differentially expressed genes in CD8^+^ T-cells following co-cultured with 3T3-L1 cells. **(I)** qRT-PCR analysis showing the mRNA expression levels of ETV family genes in CD8^+^ T cells following co-culture with 3T3-L1 cells. *n* = 3 per group. **(J)** Design of primers targeting the ETV3 binding sites within the TOX promoter region for ChIP-qPCR analysis. **(K)** Quantification of ChIP assay results showing ETV3 enrichment at specific regions of the TOX promoter. Data are presented as mean ± standard deviation. *P < 0.05; **P < 0.01; ***P < 0.001. GC, gastric cancer; POSTN, periostin; GC, gastric cancer; CAFs, cancer associated fibroblasts; NFs, normal fibroblasts; NC, negative control; qRT-PCR, quantitative Real-time polymerase chain reaction.

Considering that the AKT-NF-κB pathway represents a canonical signaling route downstream of POSTN (15), we next investigated whether POSTN overexpression in 3T3-L1 cells could activate this pathway to mediate ICAM-1 upregulation and enhanced adhesion. Our findings demonstrated a significant activation of the AKT-NF-κB axis in 3T3-L1^POSTN^ cells, as evidenced by increased phosphorylation of AKT and p65 (**Figure 5A**). Moreover, stimulation with recombinant POSTN protein similarly induced AKT-NF-κB activation in both 3T3-L1 and NF, indicating that POSTN directly triggers this signaling cascade in fibroblasts (**Figure 5B**). To further verify that ICAM-1 upregulation was mediated through the AKT-NF-κB pathway, we performed rescue experiments using the AKT inhibitor (MK2206) and the NF-κB inhibitor (JSH-23). Treatment with either inhibitor markedly attenuated ICAM-1 expression, confirming that POSTN-induced ICAM-1 activation is dependent on the AKT-NF-κB signaling cascade (**Figures 5C, D**). Although our findings support a central role for integrin/AKT/NF-κB signaling in POSTN-mediated ICAM-1 induction, additional pathways may also participate in this regulatory process within the metastatic tumor microenvironment and warrant further investigation. Consistently, blockade of the AKT-NF-κB pathway also led to a significant reduction in the adhesive ability of 3T3-L1^POSTN^ cells (**Figure 5E**).

### 3.6 ICAM-1-ETV3 signaling cascade underlies POSTN^+^ CAF-induced T-cell dysfunction

Given that exosome-derived ICAM-1 has been reported to impair T-cell activity (24), we postulated that the T-cell exhaustion observed in co-culture with 3T3-L1^POSTN^ cells might similarly be mediated via ICAM-1-dependent mechanisms. To further verify whether ICAM-1 mediates 3T3-L1^POSTN^-induced T-cell exhaustion, we treated the cells with the ICAM-1 inhibitor A-205804 (**Figure 5G**), ICAM-1-targeting siRNA (**Supplementary Figure 4H**), and the LFA-1/ICAM-1 interaction blocker A-286982 (**Figure 5G**). Following these interventions, the inhibitory effects of 3T3-L1^POSTN^ cells on CD8^+^ T-cell proliferation and effector function were markedly attenuated. To elucidate the downstream signaling pathways associated with ICAM-1-mediated T-cell dysfunction, we performed transcriptomic sequencing of CD8^+^ T-cells following co-culture with 3T3-L1^POSTN^ cells. ETV3 expression was significantly elevated in CD8^+^ T-cells after co-culture with 3T3-L1^POSTN^ cells (**Figure 5H**). Given that members of the ETV transcription factor family have been previously implicated in promoting T-cell exhaustion (25), we analyzed their expression in CD8^+^ T-cells after co-culture with 3T3-L1^POSTN^ cells. Although co-culture induced moderate upregulation of ETV1–6 in CD8^+^ T-cells (**Figure 5I**), pharmacological blockade of the LFA-1/ICAM-1 axis with A286982 selectively suppressed ETV3, highlighting its central role as a downstream effector of POSTN^+^ CAF-mediated, ICAM-1-dependent T-cell exhaustion (**Supplementary Figure 4I**). Finally, ChIP-qPCR analysis showed that ETV3 was significantly enriched at the TOX promoter relative to the control, indicating that ETV3 directly regulates TOX transcription (**Figures 5J, K**). Collectively, these results suggest that POSTN^+^ CAFs induce CD8^+^ T-cell exhaustion through an ICAM-1-ETV3 axis.

### 3.7 Combined blockade of POSTN signaling and ICIs suppresses peritoneal metastasis and reverses T-cell exhaustion in gastric cancer

**Figure 6:**
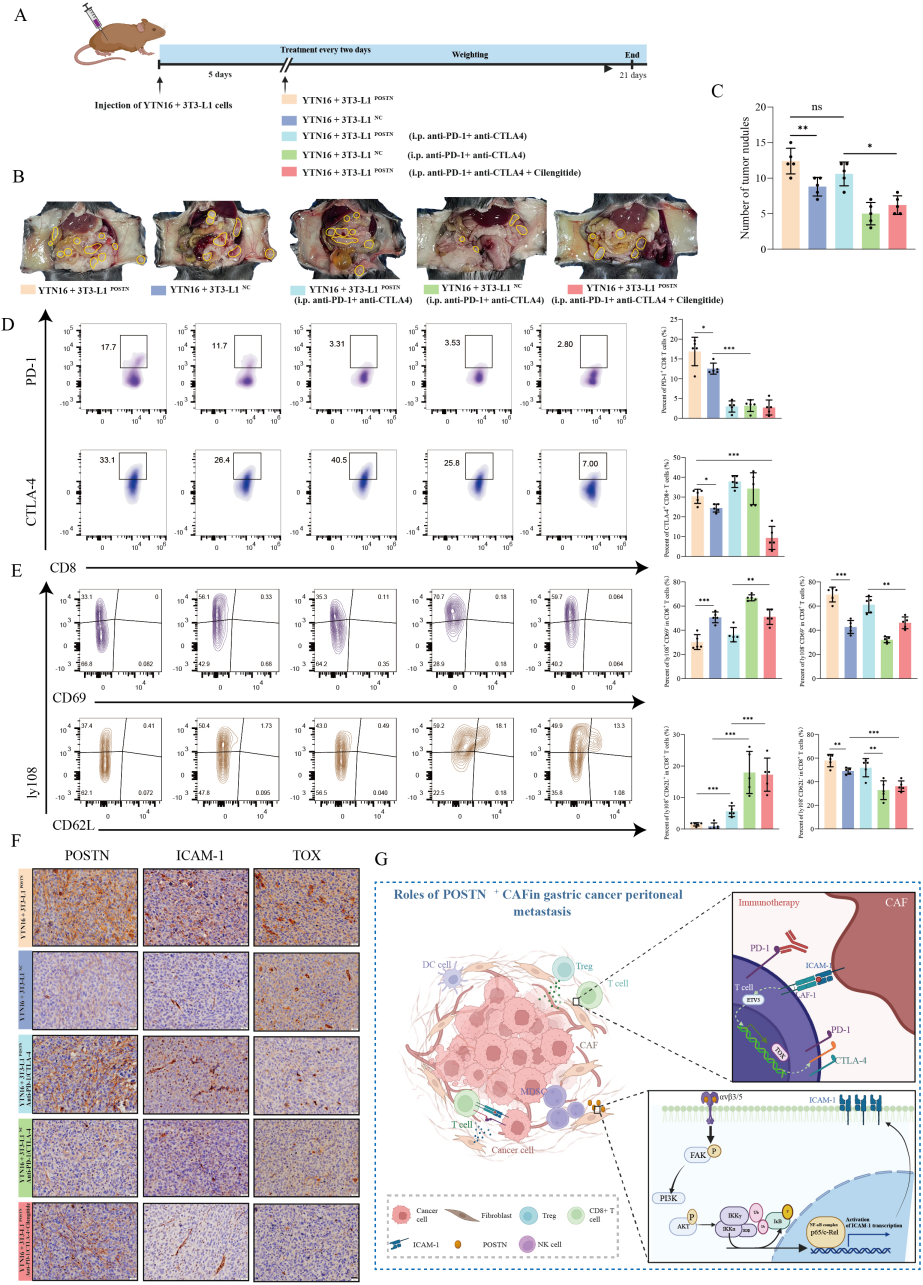
Combination therapy with anti-PD-1, anti-CTLA-4, and Cilengitide inhibits peritoneal metastasis of GC driven by POSTN^+^ CAFs. **(A)** A GCPM model was established in C57BL/6 mice by intraperitoneal injection of YTN16 cells and 3T3-L1^POSTN^ cells. Starting on day 5 post-implantation, mice were administered anti-PD-1 (10 mg/kg) and anti-CTLA-4 (10 mg/kg), with or without Cilengitide (5 mg/kg), every other day (*n* = 5 per group). **(B)** Representative images showing intraperitoneal implantation of tumors in the GCPM mouse model. **(C)** Statistical analysis of the number of peritoneal metastatic nodules in each group. **(D)** Representative flow cytometry plots showing PD-1 and CTLA-4 expression in CD8^+^ T-cells isolated from the peritoneal lavage fluid of mice. *n* = 5 per group. **(E)** Quantification of exhaustion marker expression in CD8^+^ T-cells. *n* = 5 per group. T-cell exhaustion subsets were gated and defined according to previous studies: Ly108^+^ CD69^-^ (Tex prog) and Ly108^-^ CD69^-^ (Tex int); Ly108^+^ CD62L^+^ (Tex prog), and Ly108^-^ CD62L^-^ (Tex int). **(F)** IHC analysis of POSTN, ICAM-1, and TOX expression in peritoneal tumor nodules. **(G)** Schematic diagram illustrating the molecular mechanism by which POSTN^+^ CAFs promote GCPM (drawn by BioRender). Data are presented as mean ± standard deviation. *P < 0.05; **P < 0.01; ***P < 0.001. GC, gastric cancer; GCPM, gastric cancer peritoneal metastasis; IHC, immunohistochemistry; POSTN, periostin; CAFs, cancer associated fibroblasts; NFs, normal fibroblasts; Tex prog, T cell exhaustion progenitor; Tex int, T cell exhaustion intermediate.

To determine whether targeting POSTN signaling could counteract its pro-adhesive and immunosuppressive effects and potentiate ICIs therapy in GCPM, we established a peritoneal colonization model by intraperitoneally co-injecting 3T3-L1^POSTN^ cells and YTN16 cells into C57BL/6 mice. The mice were subsequently treated with ICIs (anti-PD-1 and anti-CTLA-4) with or without the integrin antagonist Cilengitide to evaluate combinatorial therapeutic efficacy (**Figure 6A**). co-administration of ICIs and the integrin antagonist Cilengitide led to a significant reduction in peritoneal tumor implantation compared with either monotherapy or control, highlighting the synergistic potential of targeting POSTN-mediated adhesion and immune suppression in GCPM (**Figures 6B, C**). CD8^+^ T-cells in the peritoneal tumor microenvironment of mice receiving combination therapy exhibited markedly reduced expression of inhibitory receptors (**Figures 6D, F**). Importantly, combination therapy remodeled the peritoneal tumor immune landscape by elevating the proportion of progenitor (Tex prog, Ly108^+^ CD69^-^; Tex prog, Ly108^+^ CD62L^+^) CD8^+^ T-cells while reducing exhausted (Tex int, Ly108^-^ CD69^-^; Tex int, Ly108^-^ CD62L^-^) populations, indicating a partial reversal of T-cell exhaustion and enhanced potential for immune-mediated tumor control (**Figure 6F**). These results indicate that blockade of POSTN-integrin signaling in combination with ICIs effectively restores CD8^+^ T-cell function and suppresses peritoneal metastasis *in vivo*.

## 4 Discussion

PM remains one of the major determinants of poor prognosis in patients with GC, underscoring the urgent need for novel biomarkers and therapeutic targets to improve clinical outcomes (26, 27). Increasing evidence has highlighted the critical role of T-cell exhaustion in the development and progression of GCPM, as well as its close association with immune evasion and therapeutic resistance (28). However, the molecular mechanisms and key regulators driving this exhausted phenotype have not been fully elucidated. In the present study, we identified the ECM protein POSTN as a crucial mediator that is markedly upregulated in peritoneal metastatic lesions of GC. Single-cell and histological analyses revealed that POSTN is predominantly localized within CAFs, where it plays a central role in shaping the metastatic and immunosuppressive tumor microenvironment. Mechanistically, our findings suggest that POSTN^+^ CAFs exert both direct and indirect protumorigenic effects during GCPM. POSTN-mediated activation of integrin/AKT/NF-κB signaling directly enhances ICAM-1 expression and tumor cell adhesive capacity, thereby facilitating peritoneal colonization. In parallel, sustained ICAM-1/LFA-1 interactions indirectly promote CD8^+^ T-cell exhaustion through activation of exhaustion-associated transcriptional programs, ultimately contributing to reduced responsiveness to immune checkpoint blockade.

Despite the encouraging outcomes of ICIs in several clinical trials of GC, their therapeutic efficacy is substantially compromised in patients with peritoneal dissemination (29, 30). This highlights the urgent need to better predict the risk of PM and to define the subset of patients who could derive the greatest benefit from immunotherapy, thereby optimizing treatment strategies and improving overall prognosis. In recent years, comprehensive genomic and transcriptomic profiling of primary GCs has been conducted to elucidate the molecular signatures and subtypes closely associated with PM and therapeutic resistance (31). Genomic subtyping or the identification of gene panels and molecular signatures associated with a high risk of PM may substantially improve early detection and provide valuable guidance for adjuvant therapy decision-making (32–34). In our previous studies, we observed that the degree of T-cell exhaustion progressively increased with GC progression and metastasis (35, 36). Tumor-infiltrating T-cells exhibited impaired cytotoxic activity and reduced cytokine production, suggesting that T-cell exhaustion may play a critical role in driving tumor progression and metastatic dissemination in GC (21, 37). Therefore, in this study, we established a machine learning-based predictive model leveraging potential T-cell exhaustion-associated genes to stratify patients with GC according to their risk of developing PM and to infer their likely responsiveness to immunotherapy. The model, derived from a six-gene signature identified through rigorous cross-validation, exhibited robust prognostic performance in predicting survival outcomes and accurately distinguished high-risk from low-risk patient subsets. Importantly, the exhaustion-related risk score not only correlated with key immune infiltration patterns and checkpoint molecule expression but also provided novel insights into the immunological landscape underlying GC peritoneal dissemination (38, 39). Subsequent investigation of the six-gene signature revealed POSTN as a key mediator, potentially orchestrating critical processes underlying PM.

POSTN is an ECM protein that has been widely recognized for its involvement in multiple physiological and pathological processes, such as tissue repair, embryogenesis, inflammatory responses, and cancer development and progression (40–42). We identified POSTN as being predominantly localized within CAFs in GC, representing a marker enriched in a functionally activated CAF population. This finding aligns with previous studies that have highlighted the central role of POSTN^+^ CAFs in shaping the tumor microenvironment and promoting malignant progression (16, 43, 44). Importantly, POSTN expression alone may not define a completely distinct CAF lineage. Instead, POSTN-enriched CAF populations likely overlap with previously described CAF programs, particularly matrix-remodeling myCAF-like states and inflammatory CAF-associated signaling features, highlighting the functional plasticity and heterogeneity of CAFs within the gastric cancer peritoneal metastatic microenvironment. In thyroid and breast cancers (43, 45), POSTN^+^ CAFs facilitate tumor cell proliferation, invasion, and disease progression by secreting POSTN in a paracrine manner, thereby remodeling the tumor microenvironment to favor malignant growth. In GC, POSTN^+^ CAFs have been reported to facilitate tumor progression either by enhancing cancer cell stemness through POSTN secretion or by indirectly promoting an immunosuppressive microenvironment via macrophage polarization (44, 46). In this study, we explored the functional role of POSTN^+^ CAFs in promoting GC dissemination. We demonstrated that POSTN enhances ICAM-1 expression in CAFs through an autocrine signaling loop, thereby strengthening cell-cell adhesion and potentially contributing to tumor spheroid formation and peritoneal metastatic colonization. Accumulating evidence indicates that cancer cells undergoing epithelial-mesenchymal transition (EMT) generally lose their intercellular adhesion, facilitating detachment and dissemination (47, 48). Notably, peritoneal metastases are often derived from cohesive tumor cell clusters, where CAFs represent the dominant stromal population and provide structural and molecular support for metastatic outgrowth (49, 50). As a prototypical adhesion molecule, ICAM-1 has been widely implicated in tumor progression, where its increased expression strengthens intercellular adhesion and promotes metastatic spread. Taken together, our findings reveal a previously unrecognized mechanism by which POSTN^+^ CAFs promote gastric cancer peritoneal metastasis through the upregulation of ICAM-1-mediated adhesion.

In this study, we found that POSTN itself did not directly induce exhaustion in CD8^+^ T-cells, whereas POSTN^+^ CAFs markedly promoted the exhausted phenotype and suppressed the effector function of CD8^+^ T-cells. These findings suggest that POSTN^+^ CAFs may directly modulate T-cell function, thereby contributing to their dysfunctional or exhausted state. Previous studies have demonstrated that ICAM-1-mediated receptor-ligand interactions can bind to LFA-1 on T-cells, thereby attenuating CD8^+^ T-cell proliferation and cytotoxic activity and driving them toward an exhausted state (24, 51). In line with our observations, we hypothesize that POSTN^+^ CAFs may upregulate ICAM-1 expression and enhance its interaction with LFA-1 on T-cells, consequently inducing T-cell exhaustion and establishing an immunosuppressive tumor microenvironment.

Building on these findings, we further explored the downstream molecular events triggered by ICAM-1-LFA-1 signaling in CD8^+^ T-cells. Previous studies have shown that LFA-1 engagement can activate downstream signaling pathways including FAK/Src, PI3K-AKT, MAPK/ERK, and NF-κB, which are involved in T-cell activation and transcriptional reprogramming. In the context of chronic ICAM-1 stimulation within the POSTN^+^ CAF-enriched microenvironment, sustained activation of these pathways may contribute to ETV3 induction and subsequent CD8^+^ T-cell exhaustion. Our analyses revealed that ICAM-1-LFA-1 engagement markedly increased the expression of the E26 transformation-specific (ETS) family transcription factor ETV3, a key regulator implicated in the transcriptional programming of exhausted T-cells. Previous study demonstrated that ETS family member-ETV7 act as transcriptional repressors that restrain effector differentiation and sustain exhaustion-associated transcriptional programs (25). Consistent with this report, we observed that blocking ICAM-1-LFA-1 interactions attenuated the induction of exhaustion-related genes and partially restored the effector phenotype of CD8^+^ T-cells. Notably, co-administration of ICIs and the integrin receptor inhibitor Cilengitide significantly attenuated T-cell exhaustion and PM by targeting the POSTN-driven signaling cascade at its origin. This combined approach not only enhanced the antitumor immune response but also disrupted the immunosuppressive feedback loop established by POSTN^+^ CAFs within the tumor microenvironment. These results highlight the pivotal role of POSTN-mediated integrin signaling in promoting immune evasion and metastatic dissemination. Nevertheless, given that POSTN is also involved in physiological tissue repair, fibrosis, and extracellular matrix homeostasis, systemic inhibition of POSTN signaling may potentially induce off-target effects. Therefore, future translational strategies may require more selective approaches, including tumor-targeted delivery systems or microenvironment-specific therapeutic interventions, to improve safety and therapeutic specificity. Therefore, therapeutic strategies aimed at blocking the POSTN-ICAM-1 axis may represent a promising avenue to overcome immune resistance and improve the efficacy of immunotherapy in gastric cancer peritoneal metastasis.

## 5 Conclusions

In summary, our study suggests that POSTN^+^ CAFs promote gastric cancer peritoneal metastasis through two distinct mechanisms: enhancing tumor cell adhesion via ICAM-1 and inducing CD8^+^ T-cell exhaustion through the ICAM-1-ETV3 signaling axis (**Figure 6G**). Furthermore, therapeutic blockade of POSTN signaling in combination with ICIs and integrin antagonists suppresses peritoneal tumor colonization, partially restores cytotoxic T-cell function, and modulates the peritoneal immune landscape. Collectively, these findings support the involvement of an important CAF-mediated pathway of immune suppression in GCPM and highlight a potential combinatorial strategy to improve the effectiveness of immunotherapy in peritoneal metastasis.

## Data Availability

The datasets presented in this study can be found in online repositories. The names of the repository/repositories and accession number(s) can be found in the article/**Supplementary Material**.

## References

[1] Smyth EC Nilsson M Grabsch HI van Grieken NC Lordick F . Gastric cancer. Lancet. (2020) 396:635–48. doi: 10.1016/s0140-6736(20)31288-5 32861308

[2] Wei J Bu Z . Advances in gastric cancer treatment in 2024: Key breakthroughs and emerging trends. Chin J Cancer Res. (2024) 36:592–5. doi: 10.21147/j.issn.1000-9604.2024.06.02 39802898 PMC11724177

[3] Rau B Brandl A Piso P Pelz J Busch P Demtröder C . Peritoneal metastasis in gastric cancer: results from the German database. Gastric Cancer. (2020) 23:11–22. doi: 10.1007/s10120-019-00978-0 31228044

[4] Koemans WJ Lurvink RJ Grootscholten C Verhoeven RHA de Hingh IH van Sandick JW . Synchronous peritoneal metastases of gastric cancer origin: incidence, treatment and survival of a nationwide Dutch cohort. Gastric Cancer. (2021) 24:800–9. doi: 10.1007/s10120-021-01160-1 33495964

[5] Wang Y Liu Z Li W Zhang Y Pan K . Gastric cancer in China: Epidemiology, risk factors, and screening. Chin J Cancer Res. (2025) 37:937–48. doi: 10.21147/j.issn.1000-9604.2025.06.06 41523843 PMC12780785

[6] Cheng X Dai E Wu J Flores NM Chu Y Wang R . Atlas of metastatic gastric cancer links ferroptosis to disease progression and immunotherapy response. Gastroenterology. (2024) 167:1345–57. doi: 10.1053/j.gastro.2024.07.038 39097198

[7] Deng G Wang P Su R Sun X Wu Z Huang Z . SPI1(+)CD68(+) macrophages as a biomarker for gastric cancer metastasis: a rationale for combined antiangiogenic and immunotherapy strategies. J Immunother Cancer. (2024) 12(10):e009983. doi: 10.1136/jitc-2024-009983 39455096 PMC11529461

[8] Ng D Cyr D Khan S Dossa F Swallow C Kazazian K . Molecular mechanisms of metastatic peritoneal dissemination in gastric adenocarcinoma. Cancer Metastasis Rev. (2025) 44:50. doi: 10.1007/s10555-025-10265-3 40317360 PMC12049340

[9] Wang J Shao F Yu QX Ye L Wusiman D Wu R . The common hallmarks and interconnected pathways of aging, circadian rhythms, and cancer: Implications for therapeutic strategies. Res (Wash D C). (2025) 8:612. doi: 10.34133/research.0612 40046513 PMC11880593

[10] Yasuda T Wang YA . Gastric cancer immunosuppressive microenvironment heterogeneity: implications for therapy development. Trends Cancer. (2024) 10:627–42. doi: 10.1016/j.trecan.2024.03.008 38600020 PMC11292672

[11] Che K Luo Y Song X Yang Z Wang H Shi T . Macrophages reprogramming improves immunotherapy of IL-33 in peritoneal metastasis of gastric cancer. EMBO Mol Med. (2024) 16:251–66. doi: 10.1038/s44321-023-00012-y 38238529 PMC10897402

[12] Fujiwara Y Kinoshita J Shimada M Saito H Tsuji T Yamamoto D . Regulatory B cells drive immune evasion in the tumor microenvironment and are involved peritoneal metastasis in gastric cancer. Sci Rep. (2025) 15:27499. doi: 10.1038/s41598-025-11887-x 40721618 PMC12304303

[13] Li Y Jiang L Chen Y Li Y Yuan J Lu J . Specific lineage transition of tumor-associated macrophages elicits immune evasion of ascitic tumor cells in gastric cancer with peritoneal metastasis. Gastric Cancer. (2024) 27:519–38. doi: 10.1007/s10120-024-01486-6 38460015 PMC11016508

[14] Zebley CC Zehn D Gottschalk S Chi H . T cell dysfunction and therapeutic intervention in cancer. Nat Immunol. (2024) 25:1344–54. doi: 10.1038/s41590-024-01896-9 39025962 PMC11616736

[15] Dorafshan S Razmi M Safaei S Gentilin E Madjd Z Ghods R . Periostin: biology and function in cancer. Cancer Cell Int. (2022) 22:315. doi: 10.1186/s12935-022-02714-8 36224629 PMC9555118

[16] Wang H Liang Y Liu Z Zhang R Chao J Wang M . POSTN(+) cancer-associated fibroblasts determine the efficacy of immunotherapy in hepatocellular carcinoma. J Immunother Cancer. (2024) 12(7):e008721. doi: 10.1136/jitc-2023-008721 39067872 PMC11284881

[17] Chen C Guo Q Liu Y Hou Q Liao M Guo Y . Single-cell and spatial transcriptomics reveal POSTN(+) cancer-associated fibroblasts correlated with immune suppression and tumour progression in non-small cell lung cancer. Clin Transl Med. (2023) 13:e1515. doi: 10.1002/ctm2.1515 38115703 PMC10731139

[18] Wei WF Chen XJ Liang LJ Yu L Wu XG Zhou CF . Periostin(+) cancer-associated fibroblasts promote lymph node metastasis by impairing the lymphatic endothelial barriers in cervical squamous cell carcinoma. Mol Oncol. (2021) 15:210–27. doi: 10.1002/1878-0261.12837 33124726 PMC7782076

[19] Zhu F Liu X Li H Li J Liu H Wang Y . Identification of a novel ferroptosis-induced immunogenic cell death related signature based on a machine learning framework in colorectal cancer. Discov Oncol. (2025) 16:1289. doi: 10.1007/s12672-025-03147-1 40632358 PMC12240883

[20] Yan Y Shen Z Woo Y Wang D Liu X Zhang W . Potential role of EBI3 in gastric cancer and its inductive effects on T-cell exhaustion. J Am Coll Surg. (2025) 241:137–45. doi: 10.1097/xcs.0000000000001263 39699043

[21] Yan YJ Liu X Wang D Shen Z Zhang W Zhang Z . Tumor-derived EBI3 promotes CD8+ T cell exhaustion via STAT4-IL-10/CCL5 in gastric cancer. Cancer Immunol Res. (2025) 13(11):1873–86. doi: 10.1158/2326-6066.Cir-24-1228 40889276

[22] Gu X Wei H Suo C Shen S Zhu C Chen L . Itaconate promotes hepatocellular carcinoma progression by epigenetic induction of CD8(+) T-cell exhaustion. Nat Commun. (2023) 14:8154. doi: 10.1038/s41467-023-43988-4 38071226 PMC10710408

[23] Qian WJ Yan JS Gang XY Xu L Shi S Li X . Intercellular adhesion molecule-1 (ICAM-1): From molecular functions to clinical applications in cancer investigation. Biochim Biophys Acta Rev Cancer. (2024) 1879:189187. doi: 10.1016/j.bbcan.2024.189187 39317271

[24] Zhang W Zhong W Wang B Yang J Yang J Yu Z . ICAM-1-mediated adhesion is a prerequisite for exosome-induced T cell suppression. Dev Cell. (2022) 57:329–343.e7. doi: 10.1016/j.devcel.2022.01.002 35085484 PMC8881799

[25] Cheng J Xiao Y Peng T Zhang Z Qin Y Wang Y . ETV7 limits the antiviral and antitumor efficacy of CD8(+) T cells by diverting their fate toward exhaustion. Nat Cancer. (2025) 6:338–56. doi: 10.1038/s43018-024-00892-0 39805956

[26] Ajani JA D'Amico TA Bentrem DJ Chao J Cooke D Corvera C . Gastric cancer, version 2.2022, NCCN clinical practice guidelines in oncology. J Natl Compr Cancer Network JNCCN. (2022) 20:167–92. doi: 10.6004/jnccn.2022.0008 35130500

[27] Yao X Ajani JA Song S . Molecular biology and immunology of gastric cancer peritoneal metastasis. Transl Gastroenterol Hepatol. (2020) 5:57. doi: 10.21037/tgh.2020.02.08 33073052 PMC7530317

[28] Groen-van Schooten TS Franco Fernandez R van Grieken NCT Bos EN Seidel J Saris J . Mapping the complexity and diversity of tertiary lymphoid structures in primary and peritoneal metastatic gastric cancer. J Immunother Cancer. (2024) 12(7):e009243. doi: 10.1136/jitc-2024-009243 38955417 PMC11218001

[29] Lee JB Kim HR Ha SJ . Immune checkpoint inhibitors in 10 years: Contribution of basic research and clinical application in cancer immunotherapy. Immune Netw. (2022) 22:e2. doi: 10.4110/in.2022.22.e2 35291660 PMC8901707

[30] Takahashi Y Sunakawa Y Inoue E Kawabata R Ishiguro A Kito Y . Real-world effectiveness of nivolumab in advanced gastric cancer: the DELIVER trial (JACCRO GC-08). Gastric Cancer. (2022) 25:235–44. doi: 10.1007/s10120-021-01237-x 34427838

[31] Shao F Wang J Li A Wu R Chen J Tuo Z . Peripheral nerve-cancer interactions in the tumor microenvironment: A three-dimensional framework integrating mechanisms, modulators, and therapeutic strategies. Res (Wash D C). (2026) 9:1221. doi: 10.34133/research.1221 41928905 PMC13040228

[32] Higuchi Y Ogata T Nakanishi N Nishi M Sakamoto A Tsuji Y . Requirement of Cavin-2 for the expression and stability of IRβ in adequate adipocyte differentiation. Mol Metab. (2022) 55:101416. doi: 10.1016/j.molmet.2021.101416 34896640 PMC8728525

[33] Takeno A Takemasa I Seno S Yamasaki M Motoori M Miyata H . Gene expression profile prospectively predicts peritoneal relapse after curative surgery of gastric cancer. Ann Surg Oncol. (2010) 17:1033–42. doi: 10.1245/s10434-009-0854-1 20012501

[34] Kanda M Shimizu D Tanaka H Tanaka C Kobayashi D Hayashi M . Significance of SYT8 for the detection, prediction, and treatment of peritoneal metastasis from gastric cancer. Ann Surg. (2018) 267:495–503. doi: 10.1097/sla.0000000000002096 28026832

[35] Yan Y Shen Z Woo Y Wang D Liu X Zhang W . Potential role of EBI3 in gastric cancer and its inductive effects on T cell exhaustion. J Am Coll Surg. (2024) 241(2):137–45. doi: 10.1097/xcs.0000000000001263 39699043

[36] Liu X Yan Y Lu L Liu Y Ma J Wang X . Comparison of SOX and CAPOX in patients with advanced gastric cancer after laparoscopic D2 gastrectomy: A randomized controlled trial. Cancer Med. (2024) 13:e7326. doi: 10.1002/cam4.7326 38826114 PMC11145022

[37] Zhai Y Liang X Deng M . Myeloid cells meet CD8(+) T cell exhaustion in cancer: What, why and how. Chin J Cancer Res. (2024) 36:616–51. doi: 10.21147/j.issn.1000-9604.2024.06.04 39802897 PMC11724180

[38] Kang D Kim IH . Molecular mechanisms and potential rationale of immunotherapy in peritoneal metastasis of advanced gastric cancer. Biomedicines. (2022) 10(6):1376. doi: 10.3390/biomedicines10061376 35740397 PMC9220323

[39] Layug PJ Vats H Kannan K Arsenio J . Sex differences in CD8(+) T cell responses during adaptive immunity. WIREs Mech Dis. (2024) 16:e1645. doi: 10.1002/wsbm.1645 38581141

[40] Xu C Wang Z Zhang L Feng Y Lv J Wu Z . Periostin promotes the proliferation and metastasis of osteosarcoma by increasing cell survival and activates the PI3K/Akt pathway. Cancer Cell Int. (2022) 22:34. doi: 10.1186/s12935-021-02441-6 35057799 PMC8780812

[41] Wang H Yao L Chen J Li Y Su Z Liu Y . The dual role of POSTN in maintaining glioblastoma stem cells and the immunosuppressive phenotype of microglia in glioblastoma. J Exp Clin Cancer Res CR. (2024) 43:252. doi: 10.1186/s13046-024-03175-9 39227950 PMC11373117

[42] Zhu D Wang Z Chen S Li Y Kang X . Therapeutic potential of targeting the IRF2/POSTN/Notch1 axis in nucleus pulposus cells for intervertebral disc degeneration. J Neuroinflamm. (2025) 22:13. doi: 10.1186/s12974-025-03335-4 39844302 PMC11755837

[43] Jin X Deng Q Ye S Liu S Fu Y Liu Y . Cancer-associated fibroblast-derived periostin promotes papillary thyroid tumor growth through integrin-FAK-STAT3 signaling. Theranostics. (2024) 14:3014–28. doi: 10.7150/thno.94207 38773979 PMC11103496

[44] You T Tang H Wu W Gao J Li X Li N . POSTN secretion by extracellular matrix cancer-associated fibroblasts (eCAFs) correlates with poor ICB response via macrophage chemotaxis activation of Akt signaling pathway in gastric cancer. Aging Dis. (2023) 14:2177–92. doi: 10.14336/ad.2023.0503 37199594 PMC10676785

[45] Lin S Zhou M Cheng L Shuai Z Zhao M Jie R . Exploring the association of POSTN(+) cancer-associated fibroblasts with triple-negative breast cancer. Int J Biol Macromol. (2024) 268:131560. doi: 10.1016/j.ijbiomac.2024.131560 38631570

[46] Zhao Z Zhang Y Guo E Zhang Y Wang Y . Periostin secreted from podoplanin-positive cancer-associated fibroblasts promotes metastasis of gastric cancer by regulating cancer stem cells via AKT and YAP signaling pathway. Mol Carcinog. (2023) 62:685–99. doi: 10.1002/mc.23517 36785937

[47] Rakina M Kazakova A Villert A Kolomiets L Larionova I . Spheroid formation and peritoneal metastasis in ovarian cancer: The role of stromal and immune components. Int J Mol Sci. (2022) 23(11):6215. doi: 10.3390/ijms23116215 35682890 PMC9181487

[48] Al Habyan S Kalos C Szymborski J McCaffrey L . Multicellular detachment generates metastatic spheroids during intra-abdominal dissemination in epithelial ovarian cancer. Oncogene. (2018) 37:5127–35. doi: 10.1038/s41388-018-0317-x 29789717 PMC6137025

[49] Yamaguchi H Miyazaki M . Cell biology of cancer peritoneal metastasis: Multiclonal seeding and peritoneal tumor microenvironment. Cancer Sci. (2025) 116:1171–80. doi: 10.1111/cas.70021 39948828 PMC12044651

[50] Miyazaki M Nakabo A Nagano Y Nagamura Y Yanagihara K Ohki R . Tissue factor-induced fibrinogenesis mediates cancer cell clustering and multiclonal peritoneal metastasis. Cancer Lett. (2023) 553:215983. doi: 10.1016/j.canlet.2022.215983 36404569

[51] Chen M Fu Z Wu C . Tumor-derived exosomal ICAM1 promotes bone metastasis of triple-negative breast cancer by inducing CD8+ T cell exhaustion. Int J Biochem Cell Biol. (2024) 175:106637. doi: 10.1016/j.biocel.2024.106637 39147124

