# Supplementary material for: POSTN+ CAFs facilitate gastric cancer peritoneal metastasis by promoting ICAM-1-dependent tumor cell adhesion and CD8+ T-cell exhaustion
Source: Front Immunol. 2026 Jun 10;17:1796080. doi: 10.3389/fimmu.2026.1796080 (PMC13291120; doi:10.3389/fimmu.2026.1796080)
Supplement: Supplementary file 1 [file DataSheet1.docx]

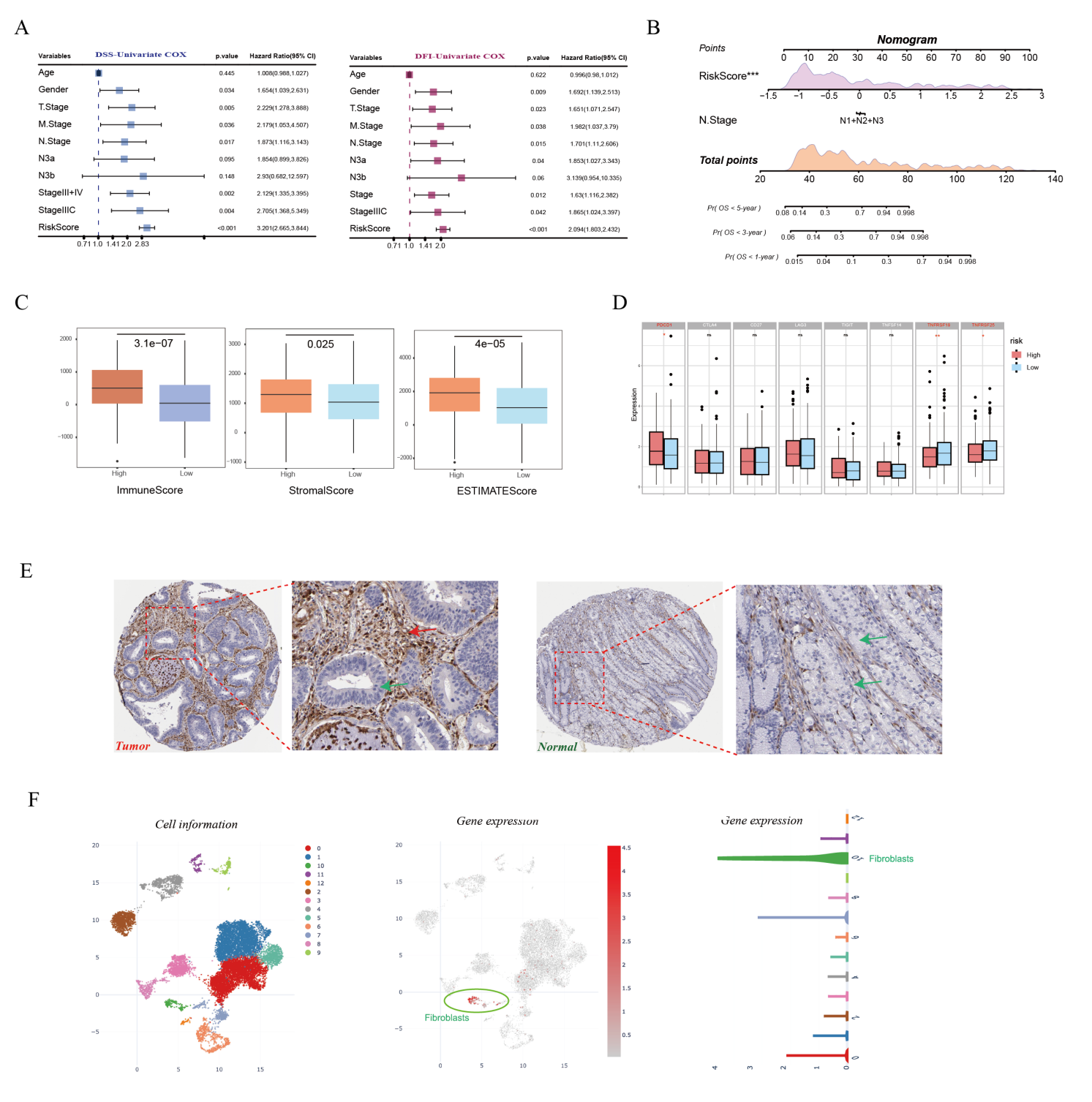


**Supplementary Figure 1.** (A) Univariate analysis of clinical characteristics along with predictive model regarding DSS and DFI. (B) A nomogram was constructed incorporating the predictive model along with relevant clinical factors, such as patient age, tumor grade, and stage. (C) Distinct immune statuses of low- and high-risk groups were assessed using immune, ESTIMATE, and stromal scores. (D) The relationship between patient risk stratification (high vs. low-risk groups) and the expression of key immune checkpoint genes was evaluated. (E) Representative IHC staining images for POSTN expression in GC and normal tissues using the HPA database. (F) Analysis of POSTN expression at the single-cell level in GCPM using data from the CDCP database. Data are presented as mean ± standard deviation. ns; not significant; **P* < 0.05; ***P* < 0.01; ****P* < 0.001. Abbreviations: WGCNA, weighted gene co-expression network analysis; DSS, disease specific survival; DFI, disease free interval; GSVA, gene set variation analysis; ESTIMATE, estimation of stromal and immune cells in malignant tumor tissues using expression data; CDCP, Cell-omics Data Coordinate Platform; GC, gastric cancer; IHC, immunohistochemistry; POSTN, periostin; CAFs, cancer associated fibroblasts; HPA, human protein atlas database; GCPM, gastric cancer peritoneal metastasis.
