# Supplementary material for: POSTN+ CAFs facilitate gastric cancer peritoneal metastasis by promoting ICAM-1-dependent tumor cell adhesion and CD8+ T-cell exhaustion
Source: Front Immunol. 2026 Jun 10;17:1796080. doi: 10.3389/fimmu.2026.1796080 (PMC13291120; doi:10.3389/fimmu.2026.1796080)
Supplement: Supplementary file 2 [file DataSheet2.docx]

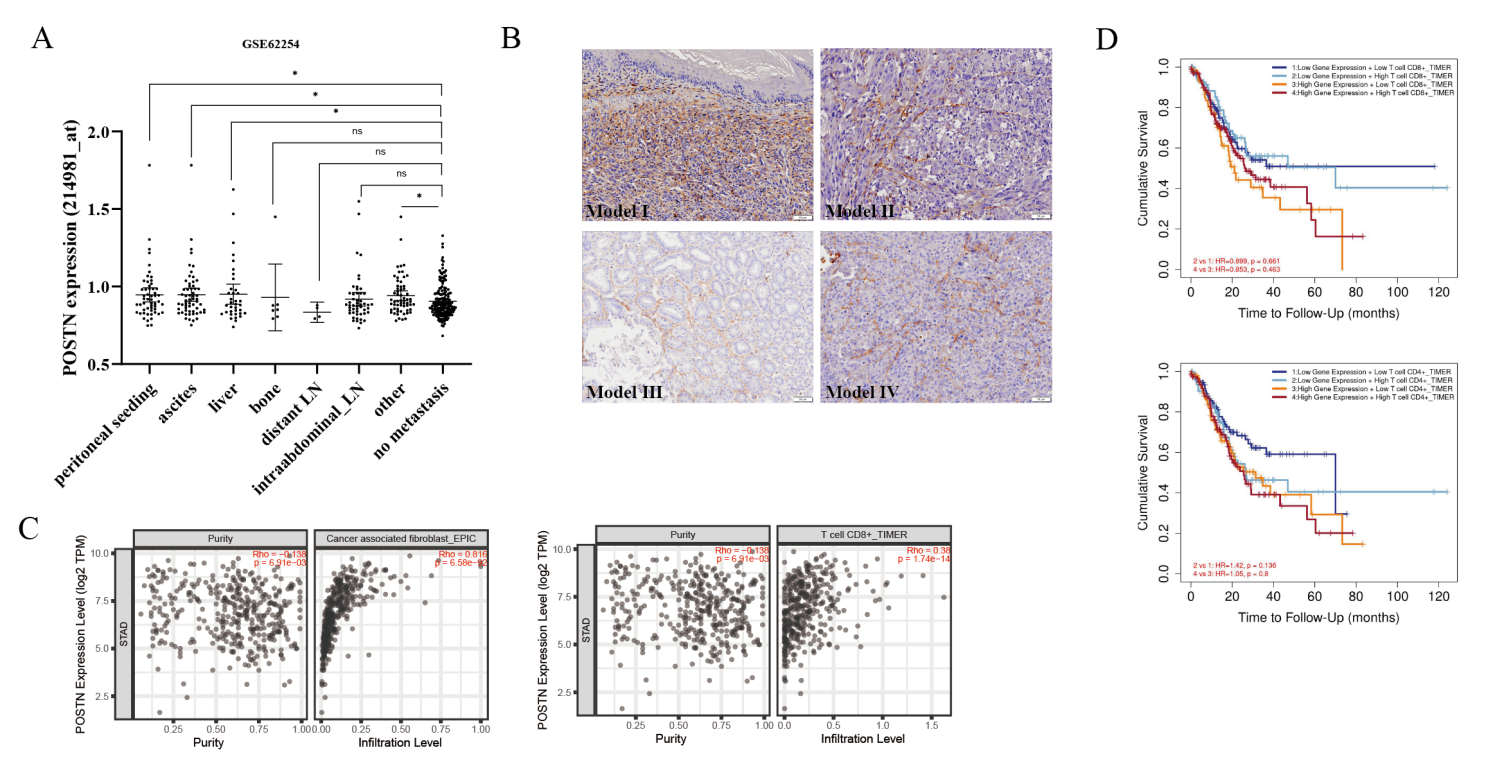


**Supplementary Figure 2.** (A) The expression levels of POSTN were assessed and compared among different metastatic lesions in GC patients (GSE62254). (B) Representative IHC staining images for POSTN expression in GCPM tissues from orthotopic GC mouse model (*n* = 4, scale bars: 50 µm). (C) Correlation between POSTN expression and the abundance of CD8⁺ T-cells and fibroblasts in GC tissues was evaluated using TIMER 2.0 database. (D) Prognostic impact of POSTN expression and infiltration of CD8⁺ and CD4⁺ T-cells in GC patients analyzed using the TIMER 2.0 database. Data are presented as mean ± standard deviation. ns; not significant; **P* < 0.05; ***P* < 0.01; ****P* < 0.001. Abbreviations:  POSTN, periostin; GC, gastric cancer; IHC, immunohistochemistry; TIMER, Tumor Immune Estimation Resource; qRT-PCR, quantitative Real-time polymerase chain reaction.
