# Supplementary material for: POSTN+ CAFs facilitate gastric cancer peritoneal metastasis by promoting ICAM-1-dependent tumor cell adhesion and CD8+ T-cell exhaustion
Source: Front Immunol. 2026 Jun 10;17:1796080. doi: 10.3389/fimmu.2026.1796080 (PMC13291120; doi:10.3389/fimmu.2026.1796080)
Supplement: Supplementary file 3 [file DataSheet3.docx]

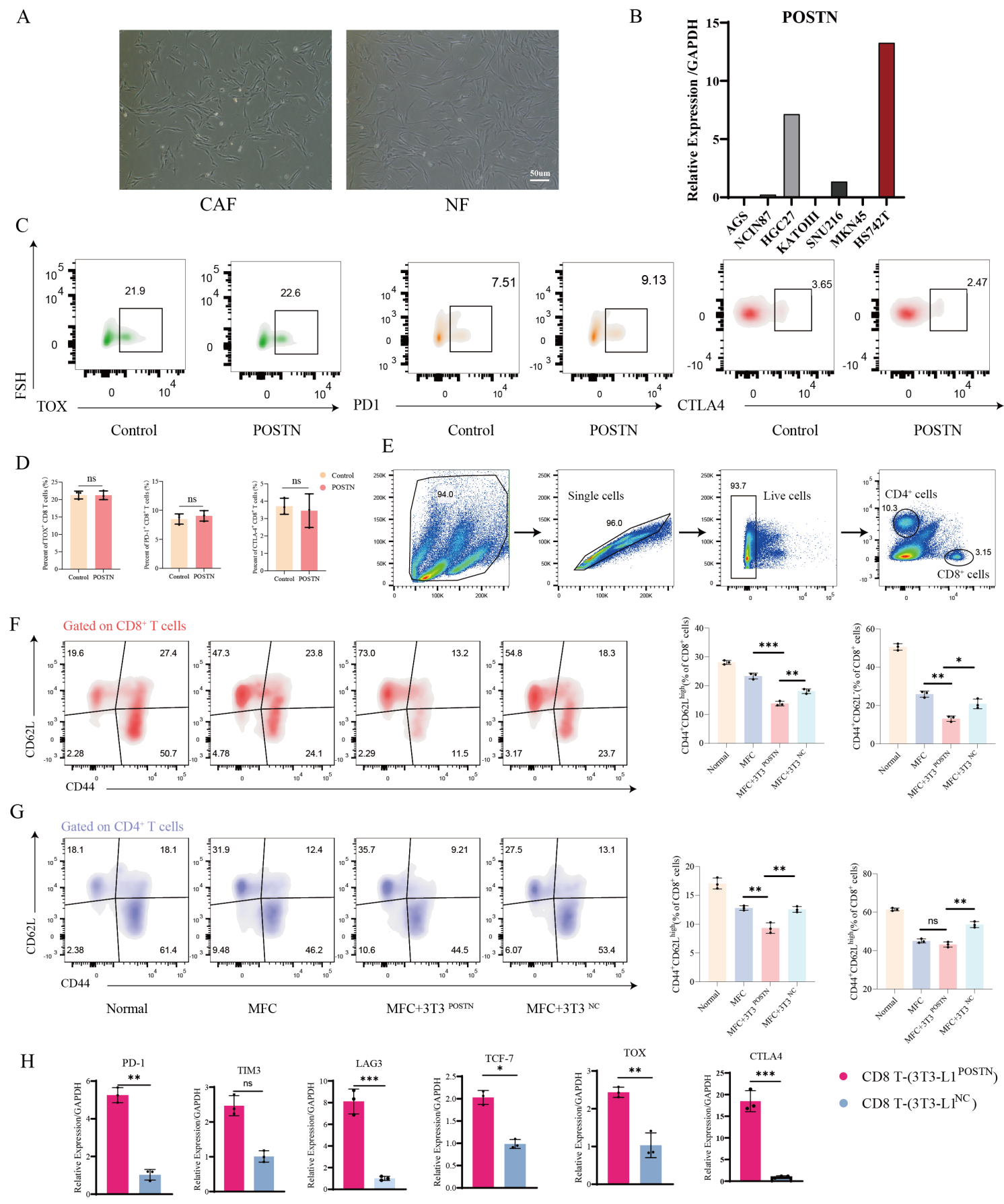


**Supplementary Figure 3.** (A) Representative microscopic images showing the morphology of primary CAFs isolated from GC tissues and NFs from adjacent non-tumor tissues (scale bars: 50 µm). (B) Analysis of POSTN expression levels in GC cell lines based on data from the CCLE database. (C-D) Representative flow cytometry plots depicting the expression of TOX, PD-1, and CTLA-4 in CD8⁺ T-cells following 72 h of POSTN treatment. (E) Flow cytometry gating strategy used for immune cell analysis in peritoneal lavage fluid. (F) Representative flow cytometry plots showing CD44 and CD62L expression in CD8⁺ T-cells isolated from the peritoneal lavage fluid of mice. Definition of T-cell subsets: Tcm (CD44⁺ CD62L⁺) and Teff (CD44^+^ CD62L⁻). (G) Representative flow cytometry plots showing CD44 and CD62L in CD4⁺ T cells isolated from the peritoneal lavage fluid of mice. Definition of T-cell subsets: Tcm (CD44⁺ CD62L⁺) and Teff (CD44⁺ CD62L⁻). (H) qRT-PCR analysis of exhaustion markers in CD8⁺ T-cells co-cultured with 3T3-L1^POSTN^ cells. *n* = 3 per group. Data are presented as mean ± standard deviation. **P* < 0.05; ***P* < 0.01; ****P* < 0.001. Abbreviations: GC, gastric cancer; CAFs, cancer associated fibroblasts; NFs, normal fibroblasts; IHC, immunohistochemistry; CCLE, Cancer Cell Line Encyclopedia; ETV, ETS variant transcription factor; Tcm, central memory T cell; Teff, effector T cell.
