# Supplementary material for: POSTN+ CAFs facilitate gastric cancer peritoneal metastasis by promoting ICAM-1-dependent tumor cell adhesion and CD8+ T-cell exhaustion
Source: Front Immunol. 2026 Jun 10;17:1796080. doi: 10.3389/fimmu.2026.1796080 (PMC13291120; doi:10.3389/fimmu.2026.1796080)
Supplement: Supplementary file 4 [file DataSheet4.docx]

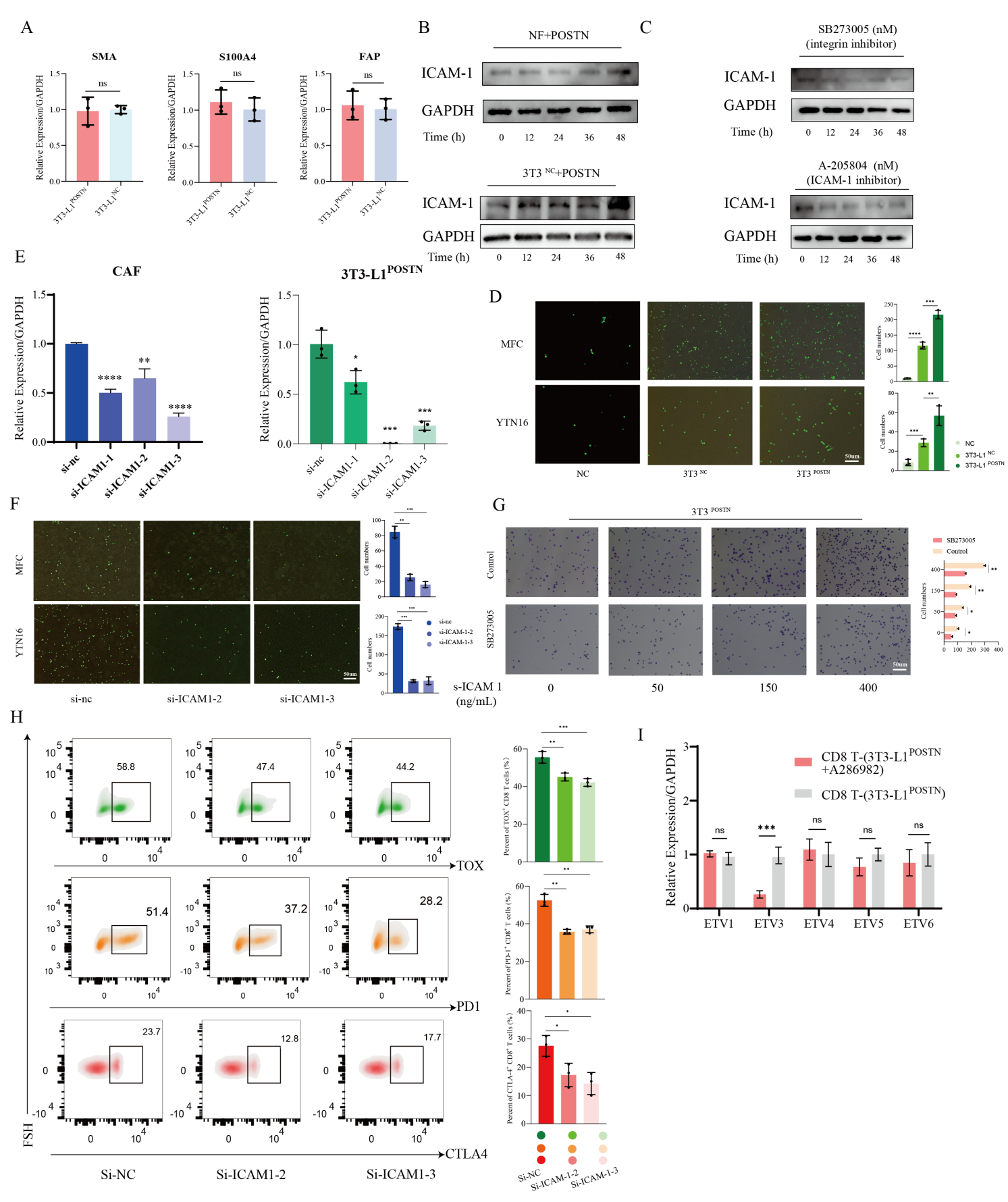


**Supplementary Figure 4.** (A) qRT-PCR analysis of SMA, S100A4, FAP expression level in 3T3-L1^POSTN^ cells. (B) Western blot analysis of ICAM-1 expression in NFs (up) and 3T3-L1^NC^ cells (down) after treatment with POSTN for different time intervals. (C) Western blot analysis of ICAM-1 expression in 3T3-L1^POSTN^ cells after treatment with SB273005 (up) and A-205804 (down) for different time intervals. (D) Adhesion assay assessing the attachment of 3T3-L1^POSTN^ cells to FITC-labeled MFC and YTN16 cells. Tumor cell suspensions were co-incubated with adherent 3T3-L1^POSTN^ cells on a shaker for 1 h to simulate peritoneal fluid motion, followed by fixation and imaging. (E) qRT-PCR analysis was performed to verify the knockdown efficiency of ICAM-1 in CAFs (left) and 3T3-L1^POSTN^ cells (right) transfected with si-ICAM-1. Three different siRNA sequences targeting ICAM-1 were designed. (F) The adhesion of 3T3-L1^POSTN^ cells to FITC-labeled MFC and YTN16 cells was evaluated after treated with si-ICAM-1. (G) Cell adhesion assay evaluating 3T3-L1 cells attachment at 50-, 60-, and 70-minutes following treatment with SB273005 and various concentrations of recombinant ICAM-1 protein. (H) Representative flow cytometry analysis of cytokines expression of TOX, PD-1, and CTLA-4 in CD8^+^ T-cells following 72 h of co-culture with 3T3-L1^POSTN^ cells treated with si-ICAM-1. CD8⁺ T cells and 3T3-L1^POSTN^ cells were directly co-cultured at a 1:1 ratio (1 × 10⁵ cells each). (I) qRT-PCR analysis showing the mRNA expression levels of ETV family genes in CD8⁺ T-cells following co-culture with 3T3-L1 cells treated with the ICAM-1 inhibitor A286982 (LFA-1/ICAM-1 inhibitor). Data are presented as mean ± standard deviation. **P* < 0.05; ***P* < 0.01; ****P* < 0.001. Abbreviations: GC, gastric cancer; CAFs, cancer associated fibroblasts; NFs, normal fibroblasts; qRT-PCR, quantitative Real-time polymerase chain reaction; FITC, fluorescein isothiocyanate; ETV, ETS variant transcription factor.
