# Supplementary material for: POSTN+ CAFs facilitate gastric cancer peritoneal metastasis by promoting ICAM-1-dependent tumor cell adhesion and CD8+ T-cell exhaustion
Source: Front Immunol. 2026 Jun 10;17:1796080. doi: 10.3389/fimmu.2026.1796080 (PMC13291120; doi:10.3389/fimmu.2026.1796080)
Supplement: Supplementary file 5 [file Table1.docx]

**Supplement Table 1. The sequence of primers and siRNAs.**

| **Gene symbol** | **Species** | **Primers** | **Sequences** |
| --- | --- | --- | --- |
| *POSTN* | Human | Forward | GACCGTGTGCTTACACAAATTG |
|  |  | Reverse | AAGTGACCGTCTCTTCCAAGG |
| *Postn* | Mouse | Forward | CCTGCCCTTATATGCTCTGCT |
|  |  | Reverse | AAACATGGTCAATAGGCATCACT |
| *Fap* | Mouse | Forward | AGCCAGGATGCCACTTCTTACT |
|  |  | Reverse | CACACTTCTTGCTCGGAGGAGA |
| *[Acta2](https://www.informatics.jax.org/marker/MGI:87909)* | Mouse | Forward | GGCTTCGCTGGTGATGATGCT |
|  |  | Reverse | GGTTCAGTGGTGCCTCTGTCAG |
| *S100a4* | Mouse | Forward | TCAGGCAAAGAGGGTGACAAGT |
|  |  | Reverse | TCATGGCAATGCAGGACAGGAA |
| *FAP* | Human | Forward | CAAAGGCTGGAGCTAAGAATCC |
|  |  | Reverse | ACTGCAAACATACTCGTTCATCA |
| *ACTA2* | Human | Forward | CTATGAGGGCTATGCCTTGCC |
|  |  | Reverse | GCTCAGCAGTAGTAACGAAGGA |
| *S100A4* | Human | Forward | GATGAGCAACTTGGACAGCAA |
|  |  | Reverse | CTGGGCTGCTTATCTGGGAAG |
| *Tcf7* | Mouse | Forward | AGCTTTCTCCACTCTACGAACA |
|  |  | Reverse | AATCCAGAGAGATCGGGGGTC |
| *Pdcd1* | Mouse | Forward | ACCCTGGTCATTCACTTGGG |
|  |  | Reverse | CATTTGCTCCCTCTGACACTG |
| *Tox* | Mouse | Forward | GCTCCCGTTCCATCCACAAA |
|  |  | Reverse | TCCCAATCTCTTGCATCACAGA |
| *Il2* | Mouse | Forward | TGAGCAGGATGGAGAATTACAGG |
|  |  | Reverse | GTCCAAGTTCATCTTCTAGGCAC |
| *ICAM1* | Human | Forward | ATGCCCAGACATCTGTGTCC |
|  |  | Reverse | GGGGTCTCTATGCCCAACAA |
| *Icam1* | Mouse | Forward | GTGATGCTCAGGTATCCATCCA |
|  |  | Reverse | CACAGTTCTCAAAGCACAGCG |
| *GAPDH* | Human | Forward | ACAACTTTGGTATCGTGGAAGG |
|  |  | Reverse | GCCATCACGCCACAGTTTC |
| *Gapdh* | Mouse | Forward | AGGTCGGTGTGAACGGATTTG |
|  |  | Reverse | TGTAGACCATGTAGTTGAGGTCA |
| *Ctla4* | Mouse | Forward | TTTTGTAGCCCTGCTCACTCT |
|  |  | Reverse | CTGAAGGTTGGGTCACCTGTA |
| *Lag3* | Mouse | Forward | CTGGGACTGCTTTGGGAAG |
|  |  | Reverse | GGTTGATGTTGCCAGATAACCC |
| *Etv1* | Mouse | Forward | TTAAGTGCAGGCGTCTTCTTC |
|  |  | Reverse | GGAGGCCATGAAAAGCCAAA |
| *Etv2* | Mouse | Forward | CTGGGAGCGGAATTTGGTTTC |
|  |  | Reverse | GTAAAGCGGGGTTCCAGTCC |

| **Gene symbol** | **Species** | **Primers** | **Sequences** |
| --- | --- | --- | --- |
| *Etv3* | Mouse | Forward | ATGAAAGCAGGCTGTAGCATC |
|  |  | Reverse | ATGGCGGAACTCTTCCTTCTG |
| *Etv4* | Mouse | Forward | CATTCCCAGATGATGTCTGCAT |
|  |  | Reverse | CCACAGTTGTAAGGCACCCC |
| *Etv5* | Mouse | Forward | TCAGTCTGATAACTTGGTGCTTC |
|  |  | Reverse | GGCTTCCTATCGTAGGCACAA |
| *Etv6* | Mouse | Forward | AGCAGGAACGAATTTCATACACG |
|  |  | Reverse | GGCAGGTGGATCGAGTCTTC |
| \| *si-ICAM1-1* \| \| --- \| | Human | — | \| GCCCAAGUUGUUGGGCAUATT \| \| --- \| |
| \| *si-ICAM1-2* \| \| --- \| | Human | — | \| \| GGUGAGGAGAGAUCACCAUTT \| \| --- \| \| \| --- \| --- \| |
| \| *si-ICAM1-3* \| \| --- \| | Human | — | \| \| CCUAUGGCAACGACUCCUUTT \| \| --- \| \| \| --- \| --- \| |
| \| *si-ICAM1-1* \| \| --- \| | Mouse | — | \| \| GGAUAUACAAGUUACAGAAGG \| \| --- \| \| \| --- \| --- \| |
| \| *si-Icam1-2* \| \| --- \| | Mouse | — | \| \| \| GAUCAGGAUAUACAAGUUACA \| \| --- \| \| \| --- \| --- \| \| \| --- \| --- \| --- \| |
| \| *si-Icam1-3* \| \| --- \| | Mouse | — | \| \| \| GGACCUUAACAGUCUACAACU \| \| --- \| \| \| --- \| --- \| \| \| --- \| --- \| --- \| |
| \| *si-Etv1-1* \| \| --- \| | Mouse | — | \| \| \| \| AGCAGUUUGUUCCAGACUAUC \| \| --- \| \| \| --- \| --- \| \| \| --- \| --- \| --- \| \| \| --- \| --- \| --- \| --- \| |
| \| *si-Etv1-2* \| \| --- \| | Mouse | — | \| \| \| \| GGCUUGGAAUGGAGUCCUACA \| \| --- \| \| \| --- \| --- \| \| \| --- \| --- \| --- \| \| \| --- \| --- \| --- \| --- \| |
| \| *si-Etv3-1* \| \| --- \| | Mouse | — | \| \| \| \| \| GGUUUACUUACAAGUUUAACU \| \| --- \| \| \| --- \| --- \| \| \| --- \| --- \| --- \| \| \| --- \| --- \| --- \| --- \| \| \| --- \| --- \| --- \| --- \| --- \| |
| \| *si-Etv3-2* \| \| --- \| | Mouse | — | \| \| \| \| \| CGAGUGGUGUGGUUCCUCAGA \| \| --- \| \| \| --- \| --- \| \| \| --- \| --- \| --- \| \| \| --- \| --- \| --- \| --- \| \| \| --- \| --- \| --- \| --- \| --- \| |
