# Supplementary material for: POSTN+ CAFs facilitate gastric cancer peritoneal metastasis by promoting ICAM-1-dependent tumor cell adhesion and CD8+ T-cell exhaustion
Source: Front Immunol. 2026 Jun 10;17:1796080. doi: 10.3389/fimmu.2026.1796080 (PMC13291120; doi:10.3389/fimmu.2026.1796080)
Supplement: Supplementary file 6 [file Table2.docx]

**Table S2. Antibodies used in this study.**

| **Antibody** | **Company** | **Application** | **Cat. No.** |
| --- | --- | --- | --- |
| α-SMA | ABclonal | WB/IHC/IF | A2235 |
| FAP | ABclonal | WB/IHC/IF | A23789 |
| POSTN | abcam | WB/IHC/IF | [AB14041](https://www.abcam.cn/products/primary-antibodies/periostin-antibody-ab14041) |
| PE-PD-1 | CST | Flow | 12-9985-82 |
| APC-INF-γ | Biolegend | Flow | 163514 |
| CD62L- PE | Invitrogen | Flow | 12-0621-81 |
| CD44-APC | MultiSciences | Flow | F1104403 |
| Percp-cy5.5-CD8 | MULTI SCIENCES | Flow | F21008A04 |
| ICAM-1 | abcam | WB/IHC | [AB171123](https://www.abcam.cn/products/primary-antibodies/icam1-antibody-1a29-ab171123) |
| FAK | Selleck | WB | F0431 |
| [FAK (phospho)](https://www.abcam.cn/products/primary-antibodies/fak-phospho-y397-antibody-ep2160y-ab81298) | abcam | WB | [AB81298](https://www.abcam.cn/products/primary-antibodies/fak-phospho-y397-antibody-ep2160y-ab81298) |
| PI3K | Selleck | WB | F0294 |
| PI3K (phospho) | abcam | WB | [AB278545](https://www.abcam.cn/products/primary-antibodies/pi-3-kinase-p85-alpha-phospho-y467-pi3-kinase-p55-phospho-y199-antibody-pi3ky458-1a11-ab278545) |
| AKT | Selleck | WB | F0004 |
| AKT (phospho) | Selleck | WB | F1644 |
| IKK | Selleck | WB | F0995 |
| IKK (phospho) | Selleck | WB | F0448 |
| IKbα | abcam | WB | [AB32518](https://www.abcam.cn/products/primary-antibodies/ikb-alpha-antibody-e130-ab32518) |
| IKbα (phospho) | abcam | WB | [AB133462](https://www.abcam.cn/products/primary-antibodies/ikb-alpha-phospho-s36-antibody-epr62352-ab133462) |
| P65 | Selleck | WB | F0006 |
| P65 (phospho) | Selleck | WB | F0155 |
| GAPDH | proteintech | WB | 60004-1 |
| Anti-CD16/CD32 | eBioscience | Flow | 14-0161-82 |
| APC-LAG3 | CST | Flow | 17-2231-82 |
| PE-TNF-α | CST | Flow | 12-7321-82 |
| FITC-CD4 | MULTI SCIENCES | Flow | F2100401 |
| MMP9 | abcam | WB | [AB283575](https://www.abcam.cn/products/primary-antibodies/mmp9-antibody-rm1020-ab283575) |
| PEcy7-CTLA4 | Invitrogen | Flow | 25-1529-42 |
| FITC-CD69 | Invitrogen | Flow | 11-0691-82 |
| APC-Ly108 | Invitrogen | Flow | 17-1508-82 |
