# Supplementary material for: POSTN+ CAFs facilitate gastric cancer peritoneal metastasis by promoting ICAM-1-dependent tumor cell adhesion and CD8+ T-cell exhaustion
Source: Front Immunol. 2026 Jun 10;17:1796080. doi: 10.3389/fimmu.2026.1796080 (PMC13291120; doi:10.3389/fimmu.2026.1796080)
Supplement: Supplementary file 7 [file Table3.docx]

**Table S3. The sequence of primers used in ChIP-qPCR.**

| **Site** | **Primers** | **Sequences** |
| --- | --- | --- |
| *1* | Forward | ACTTCCTTGCTATGCCCAGTT |
|  | Reverse | AGTTCTGTCCATAGCATGTTGA |
| *2* | Forward | AGTGTTAAGCTCTCTCGTGGT |
|  | Reverse | ACCTCTGCAAGCAACCTGAT |
| *3* | Forward | ATCAGGTTGCTTGCAGAGGT |
|  | Reverse | AGCAGAAAGATGGGCTGGTT |
| *4* | Forward | ACTTTTTATAGCCCCACCCACT |
|  | Reverse | AGGTTCAGGGCTTCATAGCAC |
| *5* | Forward | AAGCTGTGTTCTTCCTACCCA |
|  | Reverse | CATCCTGGGGTTCTCTCCTC |
| *6* | Forward | GAGAAACCTGGAAAAGGCTGC |
|  | Reverse | TGCTTCACACCTTAGGGGC |
| *7* | Forward | CAACAAAGAGAGCCCAGGGA |
|  | Reverse | AGACTCCAGGGGTGCTTAGA |
